# Network Pharmacology Study of Heat-Clearing and Detoxifying Traditional Chinese Medicine for Alzheimer's Disease

**DOI:** 10.1155/2020/7831675

**Published:** 2020-04-24

**Authors:** Hongxing Li, Xinyue Zhang, Lili Gu, Ningzi Wu, Lingxi Zhang, Jiaqi Lu, Qin Li

**Affiliations:** Key Laboratory of Neuropsychiatric Drug Research of Zhejiang Province, Institute of Materia Medica, Zhejiang Academy of Medical Sciences, Hangzhou, China

## Abstract

This study aims to explore the possible homologous mechanism of 7 frequently‐used herbs for heat-clearing and detoxification in traditional Chinese medicine (HDTCM) for treating Alzheimer's disease (AD), one of the most common types of dementia, based on network pharmacology. Herbs that satisfied the criteria of containing chlorogenic acid, relating to AD and aligning with HDTCM, were simultaneously collected to determine whether they have anti-AD effect based on a survey of the literature. Herb-ingredient-target-disease networks were constructed by collecting information from the TCMSP and GeneCards public databases. The common targets of the herbs and AD were identified for conducting a Gene Ontology (GO) analyses and a Reactome pathway enrichment analysis. The results showed that PTGS1, IL-6, CASP3, and VEGFA were the predicted key gene targets. The IL-4 and IL-13 signaling pathway, the ESR-mediated signaling pathway, and the extranuclear estrogen signaling pathway were the significant pathways associated with the 7 herbs. This study revealed that the analogous anti-AD mechanism of the 7 herbs of HDTCM may be associated with anti-inflammation, which is a common effect of the chlorogenic acid and quercetin components.

## 1. Introduction

Alzheimer's disease (AD), one of the most common neurodegenerative diseases in people over 65 years old, is characterized by neurofibrillary tangles, senile plaques, neuronal loss, and cognitive decline [1–3]. According to a report, the number of people diagnosed with AD will be no less than 130 million by 2050 [4]. However, the mechanism of AD remains unclear. More than 200 clinical trials for AD around the world have been terminated because of ineffective treatment, and of the few remaining treatments, none can completely prevent the progression of AD [5]. Thus, searching for new AD drug resources, such as traditional Chinese medicine (TCM) and natural products, is of particular importance [6].

TCM, especially the herbs and formulae, has been used for more than a few thousand years in China and other Southeast Asian countries to prevent or cure all kinds of diseases, including neurodegenerative diseases [7, 8]. Based on TCM theory, ameliorating the syndrome of hyperactivity of heart-liver fire, a typical syndrome of AD, by using heat-clearing and detoxifying traditional Chinese medicine (HDTCM) is beneficial to retard the pathological progression of AD. For example, *Huanglian-Jie-Du-Tang*, a decoction that is composed of HDTCM relevant herbs such as *Coptis chinensis*, *Cortex Phellodendri*, and *Scutellaria baicalensis*, is used for clearing heat, purging pathogenic fire, and reducing amyloid-*β* accumulation during the treatment of AD [9]. In addition, a large number of modern pharmacological studies on natural ingredients isolated from HDTCM sources (such as geniposide [10], andrographolide [11], and berberine [12]) and their effects on AD have been performed. Therefore, developing new natural medicines based on HDTCM brings hope to AD patients.

Chlorogenic acid (CGA), a polyphenol component, is a widely available component in sources of HDTCM such as *Lonicera japonica* [13]. The CGA content is used as a quality control standard for some other herbs of HDTCM and TCM formulae, such as Yinzhi detoxifying granules and Qingrejiedu oral liquid, in China. More importantly, CGA can significantly alleviate inflammation and oxidative stress, two important promoters of AD. In addition, some reports have indicated that CGA has potential neuroprotective effects. Therefore, CGA was selected as one of the main components used for screening candidate herbs.

**Figure 1:**
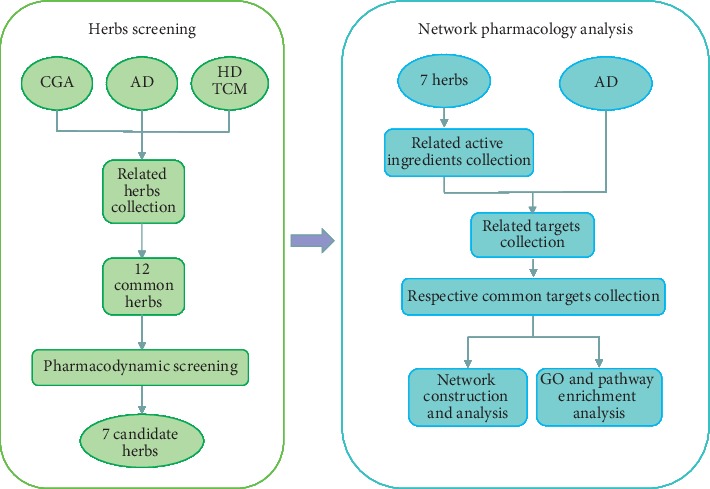
Workflow of this study.

In this study, 7 herbs were screened for further research. Currently, research on these herbs in AD treatment is limited to studies on monomer components or extracts. Network pharmacology [14, 15] is used to systematically evaluate the pharmacological effects of drugs with multiple components and multiple targets by establishing links between targets, drugs, and diseases based on the principles of systems biology. Given the characteristics of TCM and shortcomings of obsolete TCM research methods, network pharmacology was performed to explore the possible analogous mechanisms of the 7 herbs of HDTCM on AD. The workflow of this study is described in Figure 1.

## 2. Materials and Methods

### 2.1. Identification of Candidate Herbs

Herbs that satisfied the following criteria were collected, respectively: (i) contained CGA; (ii) related to AD; and (iii) aligned with HDTCM. The herbs containing CGA were collected from the Traditional Chinese Medicine Systems Pharmacology database [16] (TCMSP, http://lsp.nwu.edu.cn/tcmsp.php). The herbs relevant to AD were also collected from the TCMSP database. The classification criteria of HDTCM were based on *Chinese Pharmacopoeia 2015* (part I). All the candidate herbs were analyzed with Venny 2.1.0 (https://bioinfogp.cnb.csic.es/tools/venny/index.html) to determine the herbs that met the above criteria simultaneously. Finally, the literature was reviewed to identify the herbs have anti-AD pharmacodynamic effects.

### 2.2. Main Active Ingredient Screening and Target Collection

It was confirmed that the ADME (absorption, distribution, metabolism, and excretion) screening model is faster and more effective than other traditional models [17–19]. Therefore, the main active ingredients of these herbs were screened in the TCMSP database on the basis of two pharmacokinetic parameters: oral bioavailability (OB) and drug-likeness (DL). OB is extensively used to evaluate the ability of drugs to overcome absorption barriers and enter the blood circulation system and is determined by calculating the relationship between the drug and cytochrome P450s and P-glycoprotein [17, 20]. DL represents the ability of potential ingredients to become effective drugs by calculating the similarity with a known drug, which is beneficial to optimize pharmacokinetic properties to affect ADME [17, 21]. The active ingredients were considered viable according to ADME features with OB ≥ 30% and DL ≥ 0.18, two critical value that indicate acceptable oral bioavailability and drug-likeness as previously described [22]. All targets (including the validated and predicted targets) related to these active ingredients were extracted from the TCMSP database and entered into the UniProt database [23] (http://www.uniprot.org/) to obtain target-relevant gene names.

### 2.3. Target Fishing for AD Gene Targets

GeneCards is a comprehensive compendium of annotative information about human genes [24]. Significant AD-related genes were mainly collected from GeneCards (https://www.genecards.org/) based on a relevance score >10, a threshold that indicates a significantly high correlation with AD.

### 2.4. Network Construction and Analyses

In this study, network pharmacology was used to explore the interrelationships of the herbs, their ingredients, and targets with AD, which were represented by nodes and edges. The common targets of AD and the main active ingredients of the 7 herbs were, respectively, determined by R, a free software for statistical computing and graphic visualization. The shared targets for at least 5 herbs and AD were also processed by R, and the results were added to STRING [25] (https://string-db.org/) to construct a protein-protein-interaction (PPI) network. To comprehensively study the potential mechanism of the 7 kinds of herbs on AD, the herb-ingredient-target-disease networks and the network of herb-AD common targets were constructed by using Cytoscape 3.7.1 [26].

### 2.5. Gene Ontology and Pathway Enrichment Analyses

Gene Ontology [27] (GO), a widespread and comprehensive computational model, provides gene annotations and a logical framework of gene functions. OmicShare Tools (https://www.omicshare.com/) was utilized to perform GO enrichment analyses and thus reveal the functional changes in these targets in three respects: molecular biological function (genes that regulate molecular activity), biological process (biological programs), and cellular components (the relationship between cellular structure and gene function) [27]. Reactome [28] (http://reactome.ncpsb.org/), a visualization pathway database that highlights shared parent-child relationships among pathways to reveal underlying functional processes, was used to determine the potentially enriched pathways involved in the anti-AD effect of the 7 herbs.

## 3. Results

### 3.1. Candidate Herb Information

**Figure 2:**
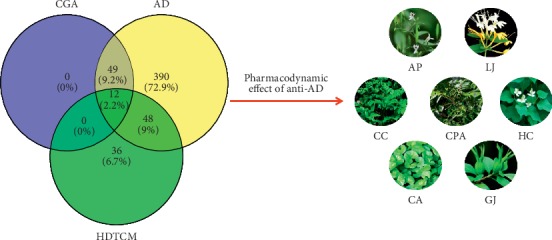
Screening process for 7 herbs.

61 herbs containing CGA and 499 herbs related to AD were collected from the TCMSP database. Furthermore, 96 herbs for which heat-clearing and detoxification effects had been clearly identified were found in the *Chinese Pharmacopoeia 2015* (part I). Then, 12 herbs that met the above criteria simultaneously emerged (Table S1). Further research based on the literature review revealed that only seven out of these twelve herbs had an anti-AD pharmacodynamic effect (Table 1). Ultimately, as shown in Figure 2, 7 herbs, namely, *Andrographis paniculata* (AP), *Coptis chinensis* (CC), *Cortex Phellodendri amurensis* (CPA), *Lonicera japonica* (LJ), *Houttuynia cordata* (HC), *Centella asiatica* (CA), and *Gardenia jasminoides* (GJ), were chosen for further study.

### 3.2. Information on the Main Active Ingredients and Targets

For each of these herbs, the main active ingredient had an OB value ≥ 30% and a DL value ≥ 0.18. Finally, a total of 64 active ingredients in these herbs were identified: 13 in AP, 10 in CC, 17 in CPA, 8 in LJ, 5 in HC, 2 in CA, and 9 in GJ. Moreover, 64 main active ingredients in the 7 herbs were associated with 1032 targets: 111 in AP, 222 in CC, 219 in CPA, 113 in LJ, 119 in HC, 80 in CA, and 168 in GJ (Tables S2–S8). Simultaneously, 711 significant gene targets linked to AD were screened out by setting the relevance score >10 (Table S9).

### 3.3. Network Construction and Analysis for the Anti-AD Targets of the 7 Herbs

First, the targets of the main active ingredients of the 7 herbs were mapped to the targets of AD. Subsequently, the herb-ingredient-target-disease networks of the 7 herbs were constructed, respectively. Among these networks, the yellow triangle nodes represent the herbs, the green ellipse nodes represent the ingredients, the blue diamond nodes represent the targets, the red rectangle nodes represent the disease, and the edges represent the interactions between each other. Degree, one of the significant topological parameters, was used to assess the importance of an ingredient or target in the network; in other words, the higher the degree, the greater the likelihood that the herb plays a role in anti-AD effect through the active ingredient or target.

**Figure 3:**
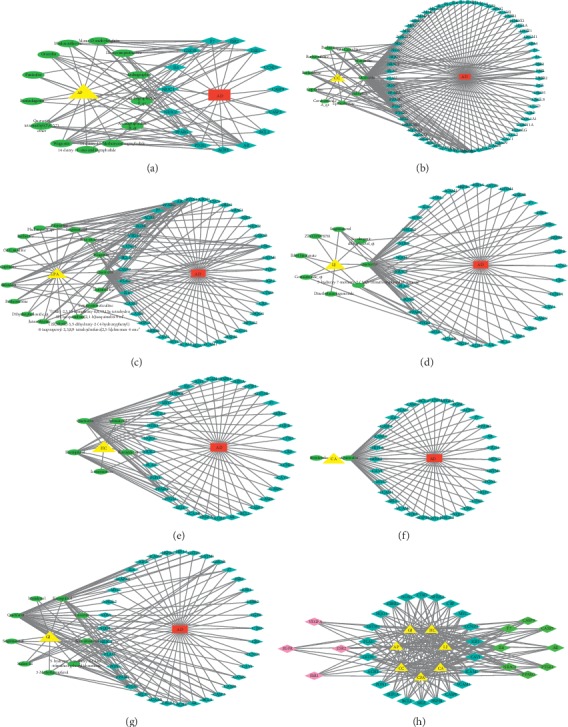
Network construction for anti-AD targets of 7 herbs. The herb-ingredient-target-disease networks of (a) AP, (b) CC, (c) CPA, (d) LJ, (e) HC, (f) CA, and (g) GJ. (h) Network of 34 shared targets for at least 5 herbs and AD (triangle: herbs; diamond: targets; purple: *n* = 5; blue: *n* = 6; green: *n* = 7).

As shown in Figure 3(a), the AP network consisted of 30 nodes (1 herb, 1 disease, 13 ingredients, and 15 targets). This network revealed that components such as wogonin (degree = 11), deoxycamptothecine (degree = 9), and quercetin tetramethyl (3′,4′,5,7) ether (degree = 9) were the high-degree ingredients and that genes such as PTGS1 (degree = 11) and AR (degree = 11) were the high-degree targets. As shown in Figure 3(b), the CC network consisted of 77 nodes (1 herb, 1 disease, 10 ingredients, and 65 targets). This network revealed that components such as quercetin (degree = 58) and palmatine (degree = 9) were the high-degree ingredients and that genes such as PTGS2 (degree = 10), AR (degree = 9), and PTGS1 (degree = 9) were the high-degree targets. As shown in Figure 3(c), the CPA network consisted of 58 nodes (1 herb, 1 disease, 17 ingredients, and 39 targets). This network revealed that components such as quercetin (degree = 33), wogonin (degree = 11), and baicalein (degree = 10) were the high-degree ingredients and that genes such as PTGS1 (degree = 15), AR (degree = 11), and ESR1 (degree = 9) were the high-degree targets. As shown in Figure 3(d), the LJ network consisted of 45 nodes (1 herb, 1 disease, 8 ingredients, and 35 targets). This network revealed that components such as quercetin (degree = 33) and 5-hydroxy-7-methoxy-2-(3,4,5-trimethoxyphenyl) chromone (degree = 7) were the high-degree ingredients and that genes such as PTGS1 (degree = 5) and AR (degree = 5) were the high-degree targets. As shown in Figure 3(e), the HC network consisted of 40 nodes (1 herb, 1 disease, 5 ingredients, and 33 targets). This network revealed that components such as quercetin (degree = 33) and kaempferol (degree = 15) were the high-degree ingredients and that genes such as NR3C2 (degree = 4) and PTGS1 (degree = 3) were the high-degree targets. As shown in Figure 3(f), the CA network consisted of 36 nodes (1 herb, 1 disease, 2 ingredients, and 32 targets). This network revealed that component quercetin (degree = 32) was the high-degree ingredients and that genes such as GSTM1 (degree = 2), PTGS1 (degree = 2), and IGF2 (degree = 2) were the high-degree targets. As shown in Figure 3(g), the GJ network consisted of 47 nodes (1 herb, 1 disease, 9 ingredients, and 36 targets). This network revealed that components such as quercetin (degree = 33) and kaempferol (degree = 15) were the high-degree ingredients and that genes such as PTGS1 (degree = 8) and PPARG (degree = 6) were the high-degree targets. Information about the ingredients and targets of the 7 herbs is illustrated in Table 2.

Based on the network constructs of the 7 herbs, 34 shared targets (Table S10) for at least 5 herbs and AD were further analyzed by R to explore analogous mechanisms of anti-AD and related biological processes among these herbs. As shown in Figure 3(h), purple represents the shared targets of 5 herbs-AD common targets (such as EGFR, ESR1, ESR2, and VEGFA), blue represents the shared targets of 6 herbs-AD common targets (such as ACHE, BCL2, CASP8, and CAV1), and green represents the shared targets of 7 herbs-AD common targets (such as AR, CASP3, CASP9, and F7).

### 3.4. PPI Network Construction and Analysis

**Figure 4:**
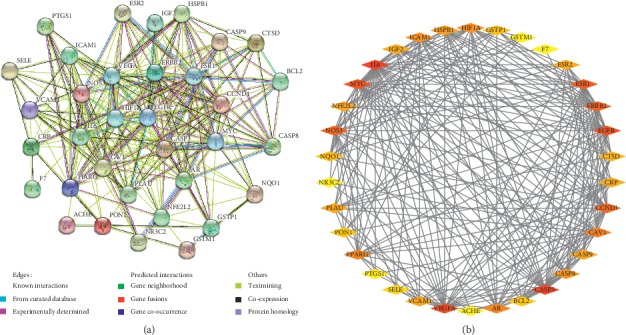
PPI network construction. (a) Nodes represent relevant targets; edges represent different interactions. (b) The darker the color, the higher the degree.

The data from thirty-four shared targets were added to STRING to construct a PPI network for exploring the interaction relationships with each other (Figure 4(a)). The degree was calculated by NetworkAnalyzer in Cytoscape to reflect the importance of a target in the network. As shown in Figure 4(b), the darker color indicates a higher degree. The analysis results showed that IL-6 (degree = 29), CASP3 (degree = 26), VEGFA (degree = 24), MYC (degree = 23), EGFR (degree = 23), ESR1 (degree = 22), and ERBB2 (degree = 21) were the pivotal targets in this network.

### 3.5. GO and Pathway Enrichment Analyses

**Figure 5:**
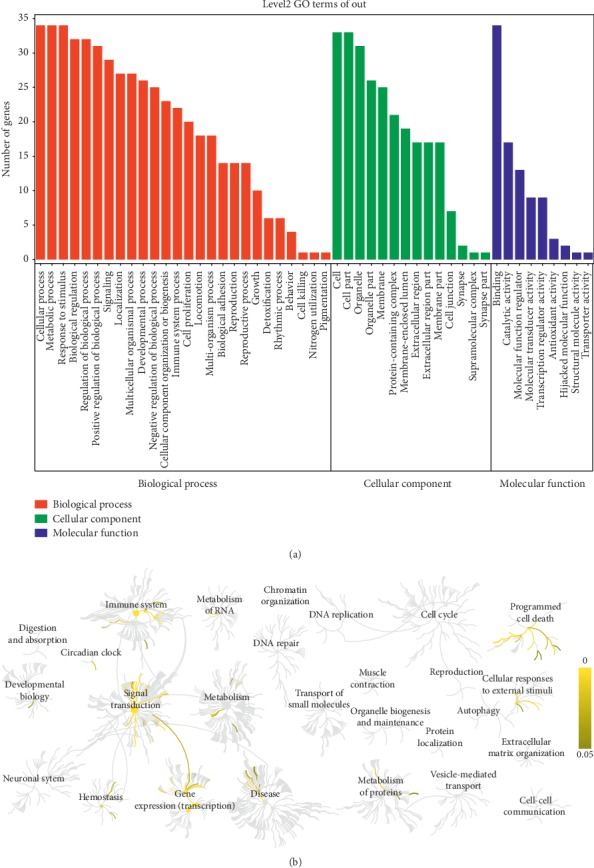
GO and pathway enrichment analysis. (a) GO analysis results of 34 common targets. (b) Reactome pathways analysis results of 34 common targets.

We carried out GO enrichment analyses to further determine the functions of these shared targets from three aspects. As shown in Figure 5(a), twenty-six functional terms were enriched in the biological process category, such as cellular process, metabolic process, response to stimulus, and biological regulation. Fourteen functional terms were enriched in the cellular components category, such as cell part and organelle part. Additionally, nine functional terms were enriched in the molecular function category, such as binding, catalytic activity, and molecular function regulator.

The pathways analysis results revealed that 33 of 34 targets were found in the Reactome database, through which 319 pathways were identified (Figure 5(b)). The enriched pathways with a *P* value ≤ 0.05 were mainly concentrated in three categories: the immune system (26/33), signal transduction (29/33), and gene expression (transcription) (22/33). Moreover, 5 remarkable leading pathways with a *P* value ≤ 0.001 are described in Table 3, as corrected by the false discovery rate (FDR), including the IL-4 and IL-13 signaling pathways; the ESR-mediated signaling pathway; the pathways of TFAP2 (AP-2) family regulating transcription of growth factors and their receptors; the extranuclear estrogen signaling pathway; and the nuclear receptors signaling pathway.

## 4. Discussion

AD is a typical neurodegenerative disease that seriously threatens human health worldwide. It is important to find new therapeutic drugs and treatment strategies for AD. In the past few decades, TCM has shown neuroprotective potential for treating AD owing to the synergistic effects of its multiple ingredients and targets [39]. HDTCM, one of the important components of TCM, has also been proven to be effective in the treatment of AD. Thus, HDTCM is expected to open a new avenue for developing drugs for the treatment of AD. Although the anti-AD efficacy of some herbs of HDTCM was confirmed, the specific mechanism had not been elucidated. Accordingly, network pharmacology was used to study the analogous mechanism of the 7 herbs of HDTCM on AD, which will improve the utilization and development of HDTCM.

In our study, 7 herbs were ultimately screened for the network pharmacology analysis. A total of 64 active ingredients, 13 in AP, 10 in CC, 17 in CPA, 8 in LJ, 5 in HC, 2 in CA, and 9 in GJ, associated with 1032 targets, 111 in AP, 222 in CC, 219 in CPA, 113 in LJ, 119 in HC, 80 in CA, and 168 in GJ, were collected to construct herb-ingredient-target-disease networks. It was found that PTGS1 (Prostaglandin Endoperoxide Synthase 1) was the high-degree target in each of the networks. The results of one analysis showed that PTGS1 is associated with arachidonic acid-induced inflammation [40]. Moreover, researchers have confirmed that the arachidonic acid metabolism pathway mediates the development of AD [41] and A*β* plaques generation [42], and the upregulation of arachidonic acid has also been observed in an AD model [43].

Furthermore, by analyzing the PPI network constructed with 34 shared targets that were regulated by these herbs, we found that IL-6 (interleukin-6), CASP3 (caspase-3), and VEGFA (vascular endothelial growth factor) were the key gene targets with the highest calculated degree. In one study, scientists reported that the level of IL-6, an inflammatory factor closely related to inflammation, in AD patients was significantly elevated [44]. Investigators have also found that downgrading the expression of CASP3 could reduce the inflammation induced by LPS in astrocytes associated with neurotic plaques in AD [45, 46]. In addition, one study found that VEGFA, an instigator of inflammation, was involved in the therapeutic regulatory network of AD and had a neuroprotective function [47].

Subsequently, the pathway analysis results showed that 34 targets were mainly enriched in pathways such as the IL-4 and IL-13 signaling pathway, the ESR-mediated signaling pathway, and the extranuclear estrogen signaling pathway. It was previously found that the IL-4 and IL-13 signaling pathways play a neuromodulating role by regulating the oxidative stress in AD and other neurodegenerative diseases [48]. Remarkably, IL-4 and IL-13 can activate microglia, the pivotal sources of inflammatory factors and oxidative stress in the brain, to induce A*β* degradation and improve cognitive impairment [49]. As implicated by increasing evidence, the regulation of neuroinflammation, one of the important incentives of AD, is a vital therapeutic strategy. Simultaneously, researchers found that the estrogen receptor can provide a protective anti-inflammatory effect by inhibiting inflammation in neurodegenerative disorders such as AD and reducing A*β* deposition in the hippocampus to improve memory capacity [50, 51].

Taken together, these results indicate that the homologous anti-AD mechanism of the 7 herbs of HDTCM may play an impotant anti-neuroinflammatory role. However, there are some factors affecting the reliability of the results, including the differences in the databases, ingredient-screening indicators, and analysis tools. Consequently, specific experimental verification is an important part of further research.

CGA has multiple biological effects as an antioxidant, antiviral, and anticarcinogenic agent. Moreover, given that CGA can pass through the blood-brain barrier and play a direct role in the central nervous system [52], new CGA studies have paid more attention to its neuroprotective effect. For example, CGA can exert antiamnesic activity by inhibiting the expression of acetylcholinesterase and malondialdehyde [53]. In addition, investigators proved the neuroprotective effects of CGA in rat cerebellar granule neurons [54]. In another interesting study, researchers found that CGA combined with selenium nanoparticles inhibited A*β* aggregation [55]. Both of these reports indicated that CGA had potential neuroprotective effects. Moreover, based on its anti-inflammatory effects, CGA is likely to be one of the main active ingredients against AD in the 7 herbs.

## 5. Conclusion

This study provides predictive insight into the mechanism of 7 frequently used herbs of HDTCM. The possible analogous anti-AD mechanism of the 7 herbs of HDTCM is related to anti-inflammation. Finally, we propose a hypothesis suggesting that CGA and quercetin, two main shared active ingredients, may play an indispensable role in the anti-AD efficacy of these 7 herbs, which requires verification and deserves further study.

## Figures and Tables

**Table 1:** Pharmacodynamic effect of anti-AD of 7 herbs.

Latin name	Chinese name	Pharmacodynamic effect of anti-AD
*Andrographis paniculata*	Chuanxinlian	Andrographolide, one of main active component of AP, reduced or eliminated A*β* aggregate and attenuated A*β* neurotoxicity [11, 29, 30]
*Coptis chinensis*	Huanglian	Berberine and polysaccharide from CC could reduce A*β*-induced toxicity and ameliorate cognitive impairment [12, 31, 32]
*Cortex Phellodendri amurensis*	Guanhuangbai	The ethanol extract of CPA has the potential protective effect against neurotoxicity induced by A*β* [33]
*Lonicera japonica*	Jinyinhua	LJ can effectively prevent the cognitive dysfunction induced by A*β* deposition [34]
*Houttuynia cordata*	Yuxingcao	HC water extract protects cortical neurons against A*β*-induced toxicity [35]
*Centella asiatica*	Jixuecao	CA extract reduces A*β* level and improves neuronal health [36, 37]
*Gardenia jasminoides*	Zhizi	GJ extract improves cognitive and memory impairment on A*β*-induced mouse [38]

**Table 2:** Information about ingredients and targets of 7 herbs.

Herbs	Numbers of active ingredients	Numbers of targets	Shared targets	High-degree ingredients	High-degree targets
AP	13	127	15	Wogonin Deoxycamptothecine Quercetin tetramethyl (3′,4′,5,7) ether	PTGS1 AR

CC	10	222	65	Quercetin palmatine	PTGS2 AR PTGS1

CPA	17	229	39	Quercetin Wogonin baicalein	PTGS1 AR ESR1

LJ	8	115	35	Quercetin 5-Hydroxy-7-methoxy-2-(3,4,5-trimethoxyphenyl) chromone	PTGS1 AR HC

HC	5	119	33	Quercetin Kaempferol	NR3C2 PTGS1

CA	2	80	32	Quercetin	GSTM1 PTGS1 IGF2

GJ	9	170	36	Quercetin Kaempferol	PTGS1 PPARG

**Table 3:** Top 5 of significant pathways.

Pathway name	*P* value	FDR	Key genes
IL-4 and IL-13 signaling	2.44*E* − 15	7.91*E* − 13	IL6; VCAM1; CCND1; MYC; BCL2; HIF1A; ICAM1; VEGFA
ESR-mediated signaling	4.41*E* − 14	7.14*E* − 12	CCND1; NOS3; CAV1; MYC; BCL2; HSPB1; CTSD; ESR1; ESR2; EGFR
TFAP2 (AP-2) family which regulates transcription of growth factors and their receptors	1.24*E* − 13	1.16*E* − 11	ERBB2; ESR1; EGFR; VEGFA
Extranuclear estrogen signaling	1.44*E* − 13	1.16*E* − 11	CCND1; NOS3; CAV1; BCL2; HSPB1; ESR1; ESR2; EGFR
Nuclear receptors signaling	1.16*E* − 12	7.44*E* − 11	CCND1; NOS3; CAV1; MYC; BCL2; HSPB1; CTSD; ESR1; ESR2; EGFR

## Data Availability

The data used to support the findings of this study are available from the corresponding author upon request.

## Supplementary Materials

Supplementary Materials Initial screening of 12 herbs (Table S1). The information about ingredients and targets of *Andrographis paniculate* (AP), *Coptis chinensis* (CC), *Cortex Phellodendri amurensis* (CPA), *Lonicera japonica* (LJ), *Houttuynia cordata* (HC), *Centella asiatica* (CA), and *Gardenia jasminoides* (GJ) (Tables S2–S8). Target information of AD (Table S9). 34 shared targets for at least 5 herbs and AD (Table S10).
