# Supplementary material for: Network Pharmacology Study of Heat-Clearing and Detoxifying Traditional Chinese Medicine for Alzheimer's Disease
Source: Evid Based Complement Alternat Med. 2020 Apr 24;2020:7831675. doi: 10.1155/2020/7831675 (PMC7196989; doi:10.1155/2020/7831675)
Supplement: Supplementary Materials — Initial screening of 12 herbs (Table S1). The information about ingredients and targets of Andrographis paniculate (AP), Coptis chinensis (CC), Cortex Phellodendri amurensis (CPA), Lonicera japonica (LJ), Houttuynia cordata (HC), Centella asiatica (CA), and Gardenia jasminoides (GJ) (Tables S2–S8). Target information of AD (Table S9). 34 shared targets for at least 5 herbs and AD (Table S10). [file 7831675.f1.doc]

TABLE S1: Initial screening of 12 herbs.

| Latin Name | Chinese Name |
| --- | --- |
| *Solidago decurrens* | *Yizhihuanghua* |
| *Coptis chinensis* | *Huanglian* |
| *Plantago depressa* | *Cheqiancao* |
| *Lonicera japonica* | *Jinyinhua* |
| *Chrysanthemum morifolium* | *Juhua* |
| *Centella asiatica* | *Jixuecao* |
| *Polygonum bistorta* | *Quanshen* |
| *Andrographis paniculata* | *Chuanxinlian* |
| *Houttuynia cordata* | *Yuxingcao* |
| *Ilex chinensis* | *Dongqing* |
| *Gardenia jasminoides* | *Zhizi* |
| *Cortex Phellodendri amurensis* | *Guanhuangbai* |

TABLE S2: The information about ingredients and targets of AP.

| Ingredients | Targets |
| --- | --- |
| Wogonin | PTGS1; ESR1; AR; PPARG; GSK3B; CHEK1; PRSS1; GABRA1; RELA; CCND1; BCL2; EIF6; CASP9; IL6; AHSA1; CASP3; TP63; TEP1; PTGER3; MCL1 |
|
|
|
|
|
| Oroxylin a | PTGS1; AR; PRSS1; NCOA1; NCOA2;  BCL2; IL6; CASP3; CCNB1; CYP2C9 |
| Panicolin | PTGS1; AR; ESR2; CHEK1; PRSS1; NCOA1 |
| 14-deoxy-11-oxo-andrographolide | NR3C2; NCOA2; NCOA1 |
| Mono-O-methylwightin | PTGS1; ESR1; AR; PPARG; F7;  CACNA2D1; ESR2; GSK3B; CHEK1;  PRSS1; NCOA2 |
| Moslosooflavone | PTGS1; AR; PPARG; ESR2; GABRA1; GSK3B; CHEK1; PRSS1; NCOA1 |
| Deoxycamptothecine | PTGS1; ESR1; AR; PPARG; F7; ACHE; ESR2; GSK3B; CHEK1; PRSS1; GABRA1; NCOA2 |
| 14-deoxy-12-methoxyandrographolide | ACHE; PGR; NR3C1; NCOA2; NCOA1 |
| Paniculogenin | NR3C2; NR3C1 |
| Andrographidine B_qt | PTGS1; AR; F7; ESR2; PRSS1 |
| Andrographin | PTGS1; AR; F7; ESR2; PRSS1;  NCOA2; NCOA1 |
| Andrographidine F_qt | PTGS1; ESR1; AR; PPARG; F7; ESR2; GSK3B; PRSS1; NCOA2; NCOA1 |
| Quercetin tetramethyl (3',4',5,7) ether | PTGS1; ESR1; AR; PPARG; F7; ACHE; ESR2; GSK3B; CHEK1; PRSS1; NCOA2 |

TABLE S3: The information about ingredients and targets of CC.

| Ingredients | Targets |
| --- | --- |
| Berberine | NOS2; PTGS1; KCNH2; ESR1; AR;  SCN5A; PTGS2; RXRA; ADRB2; PRSS1; NCOA2; PDE10A |
| Berberrubine | NOS2; PTGS1; KCNH2; ESR1; AR; SCN5A; PTGS2; RXRA; PRSS1; NCOA2 |
| Epiberberine | NOS2; KCNH2; ESR1; AR; PTGS2; RXRA; PRSS1; NCOA2; PDE10A |
| (R)-Canadine | PTGS1; CHRM3; KCNH2; CHRM1; SCN5A; CHRM5; PTGS2; HTR3A; ADRA2C; CHRM4; OPRD1; ADRA1B; SLC6A3; ADRB2; ADRA1D; SLC6A4; OPRM1; PDE10A; DRD5; RXRA; SLC6A2; ADRA1A; CHRM2 |
| Berlambine | NOS2; PTGS1; CHRM3; KCNH2; AR; SCN5A; PTGS2; F7; RXRA; ADRA1B; ADRB2; ADRA1D; PRSS1; NCOA2 |
| Corchoroside A_qt | NR3C2; NCOA2 |
| Magnograndiolide | GABRA1; GRIA2 |
| Palmatine | NOS2; PTGS1; KCNH2; ESR1; AR; SCN5A; PTGS2; RXRA; ADRB2; ESR2; PRSS1; NCOA2; F7 |
| Quercetin | PTGS1; AR; PPARG; PTGS2; NCOA2; AKR1B1; PRSS1; KCNH2; SCN5A; ADRB2; FOS; CDKN1A; EIF6; BAX; CASP9; PLAU; MMP2; MMP9; MAPK1; IL10; EGF; RB1; TNFSF15; JUN; IL6; AHSA1; CASP3; TP63; ELK1; NFKBIA; POR; ODC1; CASP8; TOP1; RAF1; SOD1; PRKCA; MMP1; HIF1A; STAT1; RUNX1T1; ERBB2; PPARG; ACACA; HMOX1; CYP3A4; CYP1A2; CAV1; MYC; F3; GJA1; CYP1A1; ICAM1; IL1B; CCL2; SELE; VCAM1; PTGER3; CXCL8; PRKCB; BIRC5; DUOX2; NOS3; HSPB1; SULT1E1; MGAM; IL2; NR1I2; CYP1B1; CCNB1; PLAT; THBD; SERPINE1; COL1A1; IFN; ALOX5IL1A; MPO; TOP2A; NCF1; ABCG2; HAS2; GSTP1; NFE2L2; NQO1; PARP1; AHR; PSMD3; SLC2A4; COL3A1; CXCL11; CXCL2; DCAF5; NR1I3; CHEK2; INSR; CLDN4; PPARA; PPARD; HSF1; CRP; CXCL1; CHUK; SPP1; RUNX2; RASSF1; E2F1; E2F2; ACPP; CTSD; IGFBP3; IGF2; CD40LG; IRF1; ERBB3; PON1; DIO1; PCOLCE; NPEPPS; HK2; RASA1; GSTM1; GSTM2 |
| Coptisine | NOS2; PTGS1; KCNH2; ESR1; AR; SCN5A; PTGS2; PRSS1 |
| Worenine | NOS2; PTGS1; ESR1; AR; PTGS2; CHEK1 |

TABLE S4: The information about ingredients and targets of CPA.

| Ingredients | Targets |
| --- | --- |
| Quercetin | PTGS1; AR; PPARG; NCOA2; AKR1B1; PRSS1; F7; ACHE; GABRA1; RELA; EGFR; VEGFA; CCND1; BCL2; FOS; EIF6; CASP9; PLAU; RB1; IL6; AHSA1; CASP; TP63; ELK1; NFKBIA; POR; CASP8; RAF1; PRKCA; HIF1A; RUNX1T1; ERBB2; PPARG; ACACA; CYP3A4; CAV1; MYC; CYP1A1; ICAM1; SELE; VCAM1; PTGER3; BIRC5; DUOX2; NOS3; HSPB1; MGAM; CYP1B1; CCNB1; ALOX5; GSTP1; NFE2L2; NQO1; PARP1; AHR; PSMD3; SLC2A4; COL3A1; DCAF5; NR1I3; CHEK2; HSF1; CRP; RUNX; RASSF1; CTSD; IGFBP3; IGF2; IRF1; ERBB3; PON1; DIO1; NPEPPS; HK2; RASA1; GSTM1; GSTM2 |
| Wogonin | PTGS1; ESR1; AR; PPARG; GSK3B; CHEK1; PRSS1; GABRA1; RELA; CCND1; BCL2; EIF6; CASP9; IL6; AHSA1; CASP3; TP63; TEP1; PTGER3; MCL1 |
| Beta-sitosterol | PGR; NCOA2; PTGS1; CHRM3; CHRM1; CHRM4; ADRA1A; CHRM2; CHRNA2; GABRA1; BCL2; CASP9; CASP3; CASP8; PRKCA; PON1 |
| Stigmasterol | PGR; NR3C2; NCOA2; NCOA1; PTGS1; AKR1B1; PLAU; CTRB1; CHRM3; CHRM1; ADRA1A; CHRM2; GABRA1 |
| Palmatine | PTGS1; ESR1; AR; ESR2;  PRSS1; NCOA2; F7 |
| Phellamurin_qt | ESR1; F7; NCOA2; NR3C1 |
| Berberine | PTGS1; ESR1; AR; PRSS1; NCOA2; PDE10A |
| (S)-Canadine | PTGS1; CHRM3; CHRM1; CHRM5; HTR3A; F7; ADRA2C; CHRM4; ADRA1A; CHRM2; DRD3; RXRB |
| Coptisine | PTGS1; ESR1; AR; PRSS1 |
| Baicalein | PTGS1; AR; PRSS1; NCOA2; NCOA1; RELA; VEGFA; BCL2; FOS; CASP3; TP63; HIF1A; FOSL1; CCNB1; AHR; IGF2; CYCS; NOX5; APOD |
| Berberrubine | PTGS1; ESR1; AR; PRSS1; NCOA2 |
| Dihydrophelloside_qt | F7 |
| Jatrorrhizine | PTGS1; ESR1; AR; PRSS1; NCOA2 |
| (2R,3R,8R)-3,5-dihydroxy-2-(4-hydroxyphenyl)  -8-isopropenyl-2,3,8,9-tetrahydrofuro[2,3-h] chromen-4-one | F7; PRSS1; NCOA2; NCOA1 |
| (13aS)-2,3,10-trimethoxy-6,8,13,13a-tetrahydro  -5H-isoquinolino[2,1-b] isoquinolin-9-ol | PTGS1; CHRM3; CHRM1; CHRM5; HTR3A; ADRA2C; CHRM4; CHRM2; DRD3; RXRB; NCOA1 |
| Tetrahydroreticuline | PTGS1; CHRM3; CHRM1; AR; PPARG; CHRM5; ADRA2C; CHRM4; GSK3B; NCOA2 |
| Thalifendine | PTGS1; ESR1; AR; PRSS1; NCOA2 |

TABLE S5: The information about ingredients and targets of LJ.

| Ingredients | Targets |
| --- | --- |
| Ethyl linolenate | PTGS1; NCOA2 |
| Secologanic dibutylacetal_qt | ESR1 |
| ZINC03978781 | PGR; NCOA2; NR3C2 |
| 5-hydroxy-7-methoxy-2-(3,4,5-trimethoxyphenyl) chromone | PTGS1; ESR1; AR; PPARG; CACNA2D1; ESR2; GSK3B; CHEK1; PRSS1; NCOA2; NCOA1 |
| Centauroside_qt | ESR1; AR; PRSS1; NCOA2; NCOA1 |
| Dinethylsecologanoside | AR |
| Stigmasterol | PGR; NR3C2; NCOA2; NCOA1; PTGS1; AKR1B1; PLAU; CTRB1; CHRM3; CHRM1; ADRA1A; CHRM2; GABRA1 |
| Quercetin | PTGS1; AR; PPARG; NCOA2; AKR1B1; PRSS1; F7; ACHE; GABRA1; RELA; EGFR; VEGFA; CCND1; BCL2; FOS; EIF6; CASP9; PLAU; RB1; IL6; AHSA1; CASP3; TP63; ELK1; NFKBIA; POR; CASP8; RAF1; PRKCA; HIF1A; RUNX1T1; ERBB2; PPARG; ACACA; CYP3A; CAV1; MYC; CYP1A1ICAM1; SELE; VCAM1; PTGER3; BIRC5; DUOX2; NOS3; HSPB1; MGAM; CYP1B1; CCNB1; ALOX5; GSTP1; NFE2L2; NQO1; PARP1; AHR; PSMD3; SLC2A4; COL3A1; DCAF5; NR1I3; CHEK2; HSF1; CRP; RUNX2; RASSF1CTSD; IGFBP3; IGF2; IRF1; ERBB3; PON1; DIO1; NPEPPS; HK2; RASA1; GSTM1; GSTM2 |

TABLE S6: The information about ingredients and targets of HC.

| Ingredients | Targets |
| --- | --- |
| Quercetin | PTGS1; AR; PPARG; NCOA2; AKR1B1; PRSS1; F7; ACHE; GABRA1; RELA; EGFR; VEGFA; CCND1; BCL2; FOS; EIF6; CASP9; PLAU; RB1; IL6; AHSA1; CASP3; TP63; ELK1; NFKBIA; POR; CASP8; RAF1; PRKCA; HIF1A; RUNX1T1; ERBB2; PPARG; ACACA; CYP3A; CAV1; MYC; CYP1A1ICAM1; SELE; VCAM1; PTGER3; BIRC5; DUOX2; NOS3; HSPB1; MGAM; CYP1B1; CCNB1; ALOX5; GSTP1; NFE2L2; NQO1; PARP1; AHR; PSMD3; SLC2A4; COL3A1; DCAF5; NR1I3; CHEK2; HSF1; CRP; RUNX2; RASSF1CTSD; IGFBP3; IGF2; IRF1; ERBB3; PON1; DIO1; NPEPPS; HK2; RASA1; GSTM1; GSTM2 |
| Kaempferol | PTGS1; AR; PPARG; NCOA2; PRSS1; PGR; CHRM1; ACHE; CHRM2; GABRA1; F7; RELA; IKBKB; BCL2; AHSA1; CASP3; MAPK8; PPARG; CYP3A4; CYP1A1; ICAM1; SELE; VCAM1; CYP1B1; ALOX5; GSTP1; AHR; PSMD; SLC2A4; NR1I3; DIO1GSTM1; GSTM2; AKR1C3 |
| Isoramanone | PGR; NR3C2; NCOA1 |
| Ruvoside_qt | NR3C2; NCOA1 |
| Spinasterol | PGR; NR3C2; NCOA2 |

TABLE S7: The information about ingredients and targets of CA.

| Ingredients | Targets |
| --- | --- |
| Quercetin | PTGS1; AR; PPARG; NCOA2; AKR1B1; PRSS1; F7; ACHE; GABRA1; RELA; EGFR; VEGFA; CCND1; BCL2; FOS; EIF6; CASP9; PLAU; RB1; IL6; AHSA1; CASP3; TP63; ELK1; NFKBIA; POR; CASP8; RAF1; PRKCA; HIF1A; RUNX1T1; ERBB2; PPARG; ACACA; CYP3A; CAV1; MYC; CYP1A1ICAM1; SELE; VCAM1; PTGER3; BIRC5; DUOX2; NOS3; HSPB1; MGAM; CYP1B1; CCNB1; ALOX5; GSTP1; NFE2L2; NQO1; PARP1; AHR; PSMD3; SLC2A4; COL3A1; DCAF5; NR1I3; CHEK2; HSF1; CRP; RUNX2; RASSF1CTSD; IGFBP3; IGF2; IRF1; ERBB3; PON1; DIO1; NPEPPS; HK2; RASA1; GSTM1; GSTM2 |
| Sitosterol | PGR; NCOA2; NR3C2 |

TABLE S8: The information about ingredients and targets of GJ.

| Ingredients | Targets |
| --- | --- |
| Quercetin | PTGS1; AR; PPARG; NCOA2; AKR1B1; PRSS1; F7; ACHE; GABRA1; RELA; EGFR; VEGFA; CCND1; BCL2; FOS; EIF6; CASP9; PLAU; RB1; IL6; AHSA1; CASP3; TP63; ELK1; NFKBIA; POR; CASP8; RAF1; PRKCA; HIF1A; RUNX1T1; ERBB2; PPARG; ACACA; CYP3A; CAV1; MYC; CYP1A1ICAM1; SELE; VCAM1; PTGER3; BIRC5; DUOX2; NOS3; HSPB1; MGAM; CYP1B1; CCNB1; ALOX5; GSTP1; NFE2L2; NQO1; PARP1; AHR; PSMD3; SLC2A4; COL3A1; DCAF5; NR1I3; CHEK2; HSF1; CRP; RUNX2; RASSF1CTSD; IGFBP3; IGF2; IRF1; ERBB3; PON1; DIO1; NPEPPS; HK2; RASA1; GSTM1; GSTM2 |
| Beta-sitosterol | PGR; NCOA2; PTGS1; CHRM3; CHRM1; CHRM4; ADRA1A; CHRM2; CHRNA2; GABRA1; BCL2; CASP9; CASP3; CASP8; PRKCA; PON1 |
| Kaempferol | PTGS1; AR; PPARG; NCOA2; PRSS1; PGR; CHRM1; ACHE; CHRM2; GABRA1; F7; RELA; IKBKB; BCL2; AHSA1; CASP3; MAPK8; PPARG; CYP3A4; CYP1A; ICAM1; SELE; VCAM1; CYP1B1; ALOX5; GSTP1; AHR; PSMD3; SLC2A4; NR1I3; DIO1GSTM1; GSTM2; AKR1C3 |
| Stigmasterol | PGR; NR3C2; NCOA2; NCOA1; PTGS1; AKR1B1; PLAU; CTRB1; CHRM3; CHRM1; ADRA1A; CHRM2; GABRA1 |
| Crocetin | CHRM3; CHRM1; ADRA1A; CHRM2; GABRA1; NCOA2; VCAM1 |
| Mandenol | PTGS1; NCOA2 |
| 5-hydroxy-7-methoxy-2-(3,4,5-trimethoxyphenyl) chromone | PTGS1; ESR1; AR; PPARG; CACNA2D1; ESR2; GSK3B; CHEK1; PRSS1; NCOA2; NCOA1 |
| Sudan III | ESR1; F7; ESR2; GSK3B; MAPK10 |
| 3-Methylkempferol | PTGS1; AR; GSK3B |

TABLE S9: Targets information of AD.

| Gene Symbol | Description | Relevance score |
| --- | --- | --- |
| APP | Amyloid Beta Precursor Protein | 158.78 |
| PSEN1 | Presenilin 1 | 143.09 |
| APOE | Apolipoprotein E | 134.78 |
| PSEN2 | Presenilin 2 | 109.82 |
| MAPT | Microtubule Associated Protein Tau | 102.93 |
| SNCA | Synuclein Alpha | 85.43 |
| TNF | Tumor Necrosis Factor | 82.99 |
| PRNP | Prion Protein | 70.76 |
| IL1B | Interleukin 1 Beta | 65.77 |
| BDNF | Brain Derived Neurotrophic Factor | 61.16 |
| NOS3 | Nitric Oxide Synthase 3 | 60.66 |
| CTSD | Cathepsin D | 58.59 |
| SORL1 | Sortilin Related Receptor 1 | 57.86 |
| BCHE | Butyrylcholinesterase | 57.26 |
| CHAT | Choline O-Acetyltransferase | 55.36 |
| ABCA1 | ATP Binding Cassette Subfamily A Member 1 | 54.29 |
| GFAP | Glial Fibrillary Acidic Protein | 54.05 |
| ADAM10 | ADAM Metallopeptidase Domain 10 | 52.45 |
| SQSTM1 | Sequestosome 1 | 52.34 |
| LRP1 | LDL Receptor Related Protein 1 | 51.37 |
| SOD1 | Superoxide Dismutase 1 | 51.11 |
| NEFL | Neurofilament Light | 50.24 |
| A2M | Alpha-2-Macroglobulin | 50.18 |
| PLAU | Plasminogen Activator, Urokinase | 49.39 |
| MPO | Myeloperoxidase | 49.16 |
| ABCA7 | ATP Binding Cassette Subfamily A Member 7 | 49.03 |
| MME | Membrane Metalloendopeptidase | 49.02 |
| PPARG | Peroxisome Proliferator Activated Receptor Gamma | 49.01 |
| FAS | Fas Cell Surface Death Receptor | 48.92 |
| TTR | Transthyretin | 48.88 |
| HFE | Homeostatic Iron Regulator | 48.78 |
| CST3 | Cystatin C | 48.26 |
| BACE1 | Beta-Secretase 1 | 48.11 |
| LRRK2 | Leucine Rich Repeat Kinase 2 | 47.84 |
| MT-ND1 | Mitochondrially Encoded NADH:Ubiquinone Oxidoreductase Core Subunit 1 | 47.79 |
| GDNF | Glial Cell Derived Neurotrophic Factor | 47.61 |
| CAT | Catalase | 47.43 |
| SLC6A4 | Solute Carrier Family 6 Member 4 | 47.06 |
| UCHL1 | Ubiquitin C-Terminal Hydrolase L1 | 47.02 |
| TARDBP | TAR DNA Binding Protein | 46.98 |
| GRN | Granulin Precursor | 46.48 |
| PRKN | Parkin RBR E3 Ubiquitin Protein Ligase | 46.47 |
| LPL | Lipoprotein Lipase | 45.97 |
| NGF | Nerve Growth Factor | 45.81 |
| HSD17B10 | Hydroxysteroid 17-Beta Dehydrogenase 10 | 45.62 |
| LDLR | Low Density Lipoprotein Receptor | 45.59 |
| ACHE | Acetylcholinesterase (Cartwright Blood Group) | 45.57 |
| MAPK1 | Mitogen-Activated Protein Kinase 1 | 45.36 |
| NCSTN | Nicastrin | 44.87 |
| TNFRSF1A | TNF Receptor Superfamily Member 1A | 44.58 |
| GBA | Glucosylceramidase Beta | 44.42 |
| SNCB | Synuclein Beta | 44.1 |
| SERPINA3 | Serpin Family A Member 3 | 43.54 |
| CLU | Clusterin | 43.37 |
| MT-ND2 | Mitochondrially Encoded NADH:Ubiquinone Oxidoreductase Core Subunit 2 | 43.33 |
| CRH | Corticotropin Releasing Hormone | 43.3 |
| HMOX1 | Heme Oxygenase 1 | 43.25 |
| ADAM17 | ADAM Metallopeptidase Domain 17 | 43.17 |
| PSENEN | Presenilin Enhancer, Gamma-Secretase Subunit | 42.92 |
| CD40 | CD40 Molecule | 42.81 |
| GRIN2B | Glutamate Ionotropic Receptor NMDA Type Subunit 2B | 42.2 |
| CASP8 | Caspase 8 | 42.08 |
| PLD3 | Phospholipase D Family Member 3 | 41.96 |
| MTHFR | Methylenetetrahydrofolate Reductase | 41.05 |
| NTRK1 | Neurotrophic Receptor Tyrosine Kinase 1 | 40.98 |
| GSK3B | Glycogen Synthase Kinase 3 Beta | 40.82 |
| PICALM | Phosphatidylinositol Binding Clathrin Assembly Protein | 40.74 |
| CTSB | Cathepsin B | 40.72 |
| RELN | Reelin | 40.7 |
| IL6 | Interleukin 6 | 40.37 |
| NTRK2 | Neurotrophic Receptor Tyrosine Kinase 2 | 40.12 |
| OLR1 | Oxidized Low Density Lipoprotein Receptor 1 | 40.09 |
| CHRNA7 | Cholinergic Receptor Nicotinic Alpha 7 Subunit | 39.61 |
| VLDLR | Very Low Density Lipoprotein Receptor | 39.47 |
| MT-CO1 | Mitochondrially Encoded Cytochrome C Oxidase I | 39.41 |
| SLC6A3 | Solute Carrier Family 6 Member 3 | 39.21 |
| DNM1L | Dynamin 1 Like | 39.18 |
| DKK1 | Dickkopf WNT Signaling Pathway Inhibitor 1 | 38.6 |
| GRIN2A | Glutamate Ionotropic Receptor NMDA Type Subunit 2A | 38.32 |
| GRIN1 | Glutamate Ionotropic Receptor NMDA Type Subunit 1 | 38.19 |
| DYRK1A | Dual Specificity Tyrosine Phosphorylation Regulated Kinase 1A | 38.18 |
| UBB | Ubiquitin B | 38.1 |
| PARK7 | Parkinsonism Associated Deglycase | 37.97 |
| COL25A1 | Collagen Type XXV Alpha 1 Chain | 37.71 |
| GAL | Galanin And GMAP Prepropeptide | 37.53 |
| DHCR24 | 24-Dehydrocholesterol Reductase | 37.39 |
| SEMA3A | Semaphorin 3A | 37.25 |
| COX5A | Cytochrome C Oxidase Subunit 5A | 37.1 |
| CAPN1 | Calpain 1 | 37.05 |
| MT-CO2 | Mitochondrially Encoded Cytochrome C Oxidase II | 36.79 |
| BPTF | Bromodomain PHD Finger Transcription Factor | 36.62 |
| MIR146A | MicroRNA 146a | 36.56 |
| SLC1A2 | Solute Carrier Family 1 Member 2 | 36.55 |
| CACNA1C | Calcium Voltage-Gated Channel Subunit Alpha1 C | 36.47 |
| IL1A | Interleukin 1 Alpha | 36.42 |
| ITM2B | Integral Membrane Protein 2B | 36.24 |
| CASP3 | Caspase 3 | 36.16 |
| ITPR1 | Inositol 1,4,5-Trisphosphate Receptor Type 1 | 36.01 |
| CDK5 | Cyclin Dependent Kinase 5 | 36 |
| MSR1 | Macrophage Scavenger Receptor 1 | 35.98 |
| TH | Tyrosine Hydroxylase | 35.8 |
| NPC1 | NPC Intracellular Cholesterol Transporter 1 | 35.21 |
| UNC5C | Unc-5 Netrin Receptor C | 34.95 |
| AD5 | Alzheimer Disease 5 | 34.32 |
| ACE | Angiotensin I Converting Enzyme | 34.23 |
| OGT | O-Linked N-Acetylglucosamine (GlcNAc) Transferase | 34.16 |
| AD6 | Alzheimer Disease 6 | 34.16 |
| PTGS2 | Prostaglandin-Endoperoxide Synthase 2 | 34.07 |
| SLC1A3 | Solute Carrier Family 1 Member 3 | 34 |
| DOCK3 | Dedicator of Cytokinesis 3 | 33.96 |
| IDE | Insulin Degrading Enzyme | 33.95 |
| TREM2 | Triggering Receptor Expressed on Myeloid Cells 2 | 33.93 |
| CAST | Calpastatin | 33.78 |
| MIR34A | MicroRNA 34a | 33.59 |
| SLC18A3 | Solute Carrier Family 18 Member A3 | 33.48 |
| MIR29A | MicroRNA 29a | 33.22 |
| LRP8 | LDL Receptor Related Protein 8 | 32.97 |
| CTNNA3 | Catenin Alpha 3 | 32.94 |
| IL10 | Interleukin 10 | 32.87 |
| PLCB1 | Phospholipase C Beta 1 | 32.78 |
| DNMBP | Dynamin Binding Protein | 32.76 |
| MAP1B | Microtubule Associated Protein 1B | 32.55 |
| PPP3CA | Protein Phosphatase 3 Catalytic Subunit Alpha | 32.53 |
| CIB1 | Calcium And Integrin Binding 1 | 32.4 |
| AD7 | Alzheimer Disease 7 | 32.36 |
| CSNK1D | Casein Kinase 1 Delta | 32.23 |
| MIR107 | MicroRNA 107 | 32.05 |
| HTR2A | 5-Hydroxytryptamine Receptor 2A | 31.79 |
| AD10 | Alzheimer Disease-10 | 31.48 |
| ITPR3 | Inositol 1,4,5-Trisphosphate Receptor Type 3 | 31.32 |
| AGER | Advanced Glycosylation End-Product Specific Receptor | 31.12 |
| AD11 | Alzheimer Disease-11 | 30.75 |
| AD12 | Alzheimer Disease 12 | 30.75 |
| AD8 | Alzheimer Disease 8 | 30.75 |
| AD17 | Alzheimer Disease 17 | 30.72 |
| MIR15A | MicroRNA 15a | 30.65 |
| AD13 | Alzheimer Disease-13 | 30.56 |
| AD14 | Alzheimer Disease 14 | 30.56 |
| AD15 | Alzheimer Disease-15 | 30.56 |
| AD16 | Alzheimer Disease 16 | 30.33 |
| MIR106B | MicroRNA 106b | 30.24 |
| MIR210 | MicroRNA 210 | 29.74 |
| COMT | Catechol-O-Methyltransferase | 29.69 |
| S100B | S100 Calcium Binding Protein B | 29.65 |
| MIR22 | MicroRNA 22 | 29.63 |
| CDK5R1 | Cyclin Dependent Kinase 5 Regulatory Subunit 1 | 29.51 |
| MAOB | Monoamine Oxidase B | 29.34 |
| MIAT | Myocardial Infarction Associated Transcript | 29.09 |
| BACE2 | Beta-Secretase 2 | 29.05 |
| TGFB1 | Transforming Growth Factor Beta 1 | 29.05 |
| MIR29B1 | MicroRNA 29b-1 | 29 |
| SYP | Synaptophysin | 28.99 |
| MIR128-1 | MicroRNA 128-1 | 28.77 |
| APBB1 | Amyloid Beta Precursor Protein Binding Family B Member 1 | 28.74 |
| MIR181C | MicroRNA 181c | 28.7 |
| PIN1 | Peptidylprolyl Cis/Trans Isomerase, NIMA-Interacting 1 | 28.62 |
| VCP | Valosin Containing Protein | 28.39 |
| AKT1 | AKT Serine/Threonine Kinase 1 | 28.33 |
| MIR320A | MicroRNA 320a | 28.31 |
| MIR197 | MicroRNA 197 | 28.04 |
| MIR93 | MicroRNA 93 | 27.98 |
| TP53 | Tumor Protein P53 | 27.82 |
| C9orf72 | C9orf72-SMCR8 Complex Subunit | 27.66 |
| CYP2D6 | Cytochrome P450 Family 2 Subfamily D Member 6 | 27.47 |
| SNCAIP | Synuclein Alpha Interacting Protein | 27.27 |
| IFNG | Interferon Gamma | 27.15 |
| MIR328 | MicroRNA 328 | 27.09 |
| NOTCH1 | Notch Receptor 1 | 26.63 |
| INS | Insulin | 26.41 |
| MIRLET7I | MicroRNA Let-7i | 26.4 |
| ATP7B | ATPase Copper Transporting Beta | 26.32 |
| NOD2 | Nucleotide Binding Oligomerization Domain Containing 2 | 26.1 |
| RPS27A | Ribosomal Protein S27a | 26.09 |
| NDUFS4 | NADH:Ubiquinone Oxidoreductase Subunit S4 | 25.72 |
| MIR298 | MicroRNA 298 | 25.7 |
| NOS1 | Nitric Oxide Synthase 1 | 25.69 |
| GAPDH | Glyceraldehyde-3-Phosphate Dehydrogenase | 25.66 |
| HLA-DRB1 | Major Histocompatibility Complex, Class II, DR Beta 1 | 25.62 |
| SLC18A2 | Solute Carrier Family 18 Member A2 | 25.59 |
| CYCS | Cytochrome C, Somatic | 25.55 |
| CALHM1 | Calcium Homeostasis Modulator 1 | 25.46 |
| VEGFA | Vascular Endothelial Growth Factor A | 25.33 |
| HMGB1 | High Mobility Group Box 1 | 24.98 |
| PTGS1 | Prostaglandin-Endoperoxide Synthase 1 | 24.92 |
| PLP1 | Proteolipid Protein 1 | 24.78 |
| GSK3A | Glycogen Synthase Kinase 3 Alpha | 24.69 |
| DPYSL2 | Dihydropyrimidinase Like 2 | 24.56 |
| EGF | Epidermal Growth Factor | 24.54 |
| GAA | Glucosidase Alpha, Acid | 24.53 |
| F2 | Coagulation Factor II, Thrombin | 24.47 |
| PINK1 | PTEN Induced Kinase 1 | 24.1 |
| CRP | C-Reactive Protein | 24.03 |
| NPC2 | NPC Intracellular Cholesterol Transporter 2 | 23.91 |
| LMNA | Lamin A/C | 23.73 |
| APLP2 | Amyloid Beta Precursor Like Protein 2 | 23.65 |
| APOC1 | Apolipoprotein C1 | 23.59 |
| VWF | Von Willebrand Factor | 23.52 |
| UBQLN1 | Ubiquilin 1 | 23.49 |
| NOTCH3 | Notch Receptor 3 | 23.48 |
| PKD1 | Polycystin 1, Transient Receptor Potential Channel Interacting | 23.46 |
| NQO1 | NAD(P)H Quinone Dehydrogenase 1 | 23.43 |
| RET | Ret Proto-Oncogene | 23.41 |
| MAPK8 | Mitogen-Activated Protein Kinase 8 | 23.37 |
| CREB1 | CAMP Responsive Element Binding Protein 1 | 23.36 |
| MAPK3 | Mitogen-Activated Protein Kinase 3 | 23.31 |
| CDKN2B-AS1 | CDKN2B Antisense RNA 1 | 23.31 |
| NGFR | Nerve Growth Factor Receptor | 23.26 |
| CETP | Cholesteryl Ester Transfer Protein | 23.18 |
| EIF2AK2 | Eukaryotic Translation Initiation Factor 2 Alpha Kinase 2 | 23.17 |
| CYP46A1 | Cytochrome P450 Family 46 Subfamily A Member 1 | 23.01 |
| TLR4 | Toll Like Receptor 4 | 22.78 |
| CCL2 | C-C Motif Chemokine Ligand 2 | 22.67 |
| MT3 | Metallothionein 3 | 22.58 |
| CASP9 | Caspase 9 | 22.45 |
| COL4A1 | Collagen Type IV Alpha 1 Chain | 22.44 |
| SNCG | Synuclein Gamma | 22.35 |
| MAP2 | Microtubule Associated Protein 2 | 22.35 |
| APH1A | Aph-1 Homolog A, Gamma-Secretase Subunit | 22.31 |
| DBN1 | Drebrin 1 | 22.31 |
| ATXN3 | Ataxin 3 | 22.24 |
| PON1 | Paraoxonase 1 | 22.2 |
| PCDH11X | Protocadherin 11 X-Linked | 22.08 |
| APBA1 | Amyloid Beta Precursor Protein Binding Family A Member 1 | 22.03 |
| CASP2 | Caspase 2 | 21.93 |
| APBA2 | Amyloid Beta Precursor Protein Binding Family A Member 2 | 21.82 |
| RYR1 | Ryanodine Receptor 1 | 21.73 |
| ATXN2 | Ataxin 2 | 21.72 |
| TF | Transferrin | 21.58 |
| NTF3 | Neurotrophin 3 | 21.52 |
| APOA1 | Apolipoprotein A1 | 21.47 |
| MPZ | Myelin Protein Zero | 21.36 |
| SLC2A1 | Solute Carrier Family 2 Member 1 | 21.35 |
| FRMD4A | FERM Domain Containing 4A | 21.3 |
| TFCP2 | Transcription Factor CP2 | 21.26 |
| DRD2 | Dopamine Receptor D2 | 21.21 |
| HTRA1 | HtrA Serine Peptidase 1 | 21.07 |
| TOMM40 | Translocase of Outer Mitochondrial Membrane 40 | 21 |
| RCAN1 | Regulator of Calcineurin 1 | 20.95 |
| MAOA | Monoamine Oxidase A | 20.95 |
| JAG1 | Jagged Canonical Notch Ligand 1 | 20.94 |
| APBA3 | Amyloid Beta Precursor Protein Binding Family A Member 3 | 20.92 |
| APOD | Apolipoprotein D | 20.89 |
| CASP7 | Caspase 7 | 20.89 |
| SST | Somatostatin | 20.88 |
| STH | Saitohin | 20.76 |
| ATP13A2 | ATPase Cation Transporting 13A2 | 20.73 |
| CLSTN1 | Calsyntenin 1 | 20.67 |
| VPS26A | VPS26, Retromer Complex Component A | 20.66 |
| APOB | Apolipoprotein B | 20.61 |
| ALB | Albumin | 20.56 |
| DRD3 | Dopamine Receptor D3 | 20.53 |
| ABCA4 | ATP Binding Cassette Subfamily A Member 4 | 20.5 |
| TFAM | Transcription Factor A, Mitochondrial | 20.41 |
| ESR1 | Estrogen Receptor 1 | 20.41 |
| PTPA | Protein Phosphatase 2 Phosphatase Activator | 20.31 |
| NHLRC1 | NHL Repeat Containing E3 Ubiquitin Protein Ligase 1 | 20.28 |
| APBB3 | Amyloid Beta Precursor Protein Binding Family B Member 3 | 20.23 |
| HTT | Huntingtin | 20.14 |
| VSNL1 | Visinin Like 1 | 20.11 |
| C4A | Complement C4A (Rodgers Blood Group) | 20.06 |
| EXOC3L2 | Exocyst Complex Component 3 Like 2 | 20.06 |
| IGF1 | Insulin Like Growth Factor 1 | 20.04 |
| MARK1 | Microtubule Affinity Regulating Kinase 1 | 20.02 |
| APLP1 | Amyloid Beta Precursor Like Protein 1 | 20 |
| FUS | FUS RNA Binding Protein | 19.86 |
| GATA1 | GATA Binding Protein 1 | 19.73 |
| GRIN2C | Glutamate Ionotropic Receptor NMDA Type Subunit 2C | 19.67 |
| ATP2A2 | ATPase Sarcoplasmic/Endoplasmic Reticulum Ca2+ Transporting 2 | 19.64 |
| CAPN2 | Calpain 2 | 19.62 |
| NRGN | Neurogranin | 19.62 |
| TBK1 | TANK Binding Kinase 1 | 19.61 |
| APBB2 | Amyloid Beta Precursor Protein Binding Family B Member 2 | 19.56 |
| MARK4 | Microtubule Affinity Regulating Kinase 4 | 19.55 |
| ACTC1 | Actin Alpha Cardiac Muscle 1 | 19.48 |
| EGFR | Epidermal Growth Factor Receptor | 19.46 |
| BLMH | Bleomycin Hydrolase | 19.46 |
| DCHS2 | Dachsous Cadherin-Related 2 | 19.42 |
| MMP9 | Matrix Metallopeptidase 9 | 19.39 |
| GJB1 | Gap Junction Protein Beta 1 | 19.39 |
| GRK2 | G Protein-Coupled Receptor Kinase 2 | 19.36 |
| ARSA | Arylsulfatase A | 19.31 |
| GSAP | Gamma-Secretase Activating Protein | 19.29 |
| CXCL8 | C-X-C Motif Chemokine Ligand 8 | 19.27 |
| GAP43 | Growth Associated Protein 43 | 19.26 |
| SLC17A5 | Solute Carrier Family 17 Member 5 | 19.24 |
| APAF1 | Apoptotic Peptidase Activating Factor 1 | 19.22 |
| IL1RN | Interleukin 1 Receptor Antagonist | 19.19 |
| PLA2G6 | Phospholipase A2 Group VI | 19.18 |
| NAE1 | NEDD8 Activating Enzyme E1 Subunit 1 | 19.16 |
| GRIA1 | Glutamate Ionotropic Receptor AMPA Type Subunit 1 | 19.14 |
| HLA-DQB1 | Major Histocompatibility Complex, Class II, DQ Beta 1 | 19.09 |
| SMPD1 | Sphingomyelin Phosphodiesterase 1 | 19.03 |
| CDKN2A | Cyclin Dependent Kinase Inhibitor 2A | 18.97 |
| TTBK1 | Tau Tubulin Kinase 1 | 18.95 |
| CTNNB1 | Catenin Beta 1 | 18.94 |
| SLC30A6 | Solute Carrier Family 30 Member 6 | 18.94 |
| TLR2 | Toll Like Receptor 2 | 18.93 |
| GLRX | Glutaredoxin | 18.91 |
| DLST | Dihydrolipoamide S-Succinyltransferase | 18.9 |
| GPC1 | Glypican 1 | 18.87 |
| HLA-B | Major Histocompatibility Complex, Class I, B | 18.71 |
| HCRT | Hypocretin Neuropeptide Precursor | 18.59 |
| ICAM1 | Intercellular Adhesion Molecule 1 | 18.55 |
| STAT3 | Signal Transducer and Activator Of Transcription 3 | 18.55 |
| MEOX2 | Mesenchyme Homeobox 2 | 18.52 |
| RTN4 | Reticulon 4 | 18.49 |
| SORCS3 | Sortilin Related VPS10 Domain Containing Receptor 3 | 18.48 |
| REG1A | Regenerating Family Member 1 Alpha | 18.41 |
| STMN2 | Stathmin 2 | 18.41 |
| SLC39A1 | Solute Carrier Family 39 Member 1 | 18.39 |
| ERBB2 | Erb-B2 Receptor Tyrosine Kinase 2 | 18.38 |
| PCSK1N | Proprotein Convertase Subtilisin/Kexin Type 1 Inhibitor | 18.38 |
| VDR | Vitamin D Receptor | 18.36 |
| TMED10 | Transmembrane P24 Trafficking Protein 10 | 18.34 |
| PITRM1 | Pitrilysin Metallopeptidase 1 | 18.32 |
| BACE1-AS | BACE1 Antisense RNA | 18.3 |
| CHI3L1 | Chitinase 3 Like 1 | 18.27 |
| DLG4 | Discs Large MAGUK Scaffold Protein 4 | 18.24 |
| EDNRB | Endothelin Receptor Type B | 18.24 |
| SERPINE1 | Serpin Family E Member 1 | 18.22 |
| GPR3 | G Protein-Coupled Receptor 3 | 18.17 |
| MMP3 | Matrix Metallopeptidase 3 | 18.09 |
| TMEM106B | Transmembrane Protein 106B | 18.08 |
| APH1B | Aph-1 Homolog B, Gamma-Secretase Subunit | 18.07 |
| KCNIP3 | Potassium Voltage-Gated Channel Interacting Protein 3 | 18.05 |
| TNFRSF11B | TNF Receptor Superfamily Member 11b | 18.05 |
| ANKS1B | Ankyrin Repeat and Sterile Alpha Motif Domain Containing 1B | 17.99 |
| EPM2A | EPM2A Glucan Phosphatase, Laforin | 17.98 |
| AATF | Apoptosis Antagonizing Transcription Factor | 17.93 |
| KRAS | KRAS Proto-Oncogene, GTPase | 17.91 |
| RYR3 | Ryanodine Receptor 3 | 17.87 |
| ERN1 | Endoplasmic Reticulum To Nucleus Signaling 1 | 17.8 |
| RTN3 | Reticulon 3 | 17.75 |
| TYMS | Thymidylate Synthetase | 17.74 |
| PADI2 | Peptidyl Arginine Deiminase 2 | 17.73 |
| ATP5PD | ATP Synthase Peripheral Stalk Subunit D | 17.69 |
| HSPB8 | Heat Shock Protein Family B (Small) Member 8 | 17.65 |
| CCR5 | C-C Motif Chemokine Receptor 5 (Gene/Pseudogene) | 17.6 |
| ERBB4 | Erb-B2 Receptor Tyrosine Kinase 4 | 17.6 |
| TM2D1 | TM2 Domain Containing 1 | 17.56 |
| DNMT1 | DNA Methyltransferase 1 | 17.5 |
| IL2RA | Interleukin 2 Receptor Subunit Alpha | 17.48 |
| LEP | Leptin | 17.47 |
| MECP2 | Methyl-CpG Binding Protein 2 | 17.47 |
| DRD4 | Dopamine Receptor D4 | 17.39 |
| AFP | Alpha Fetoprotein | 17.32 |
| MMP2 | Matrix Metallopeptidase 2 | 17.26 |
| HNRNPA2B1 | Heterogeneous Nuclear Ribonucleoprotein A2/B1 | 17.26 |
| DBH | Dopamine Beta-Hydroxylase | 17.23 |
| KLK8 | Kallikrein Related Peptidase 8 | 17.2 |
| HNRNPA1 | Heterogeneous Nuclear Ribonucleoprotein A1 | 17.16 |
| COX4I1 | Cytochrome C Oxidase Subunit 4I1 | 17.15 |
| MFN2 | Mitofusin 2 | 17.12 |
| TREX1 | Three Prime Repair Exonuclease 1 | 17.11 |
| FPR2 | Formyl Peptide Receptor 2 | 17.06 |
| CYBB | Cytochrome B-245 Beta Chain | 17.01 |
| EDN1 | Endothelin 1 | 16.93 |
| NRG1 | Neuregulin 1 | 16.9 |
| G6PC | Glucose-6-Phosphatase Catalytic Subunit | 16.85 |
| TNFRSF21 | TNF Receptor Superfamily Member 21 | 16.79 |
| NOS2 | Nitric Oxide Synthase 2 | 16.77 |
| IL4 | Interleukin 4 | 16.77 |
| VHL | Von Hippel-Lindau Tumor Suppressor | 16.66 |
| NEU1 | Neuraminidase 1 | 16.63 |
| HLA-A | Major Histocompatibility Complex, Class I, A | 16.55 |
| NPY | Neuropeptide Y | 16.5 |
| ABCB1 | ATP Binding Cassette Subfamily B Member 1 | 16.49 |
| CD40LG | CD40 Ligand | 16.37 |
| TBP | TATA-Box Binding Protein | 16.36 |
| VPS35 | VPS35 Retromer Complex Component | 16.35 |
| PTEN | Phosphatase and Tensin Homolog | 16.28 |
| CHGA | Chromogranin A | 16.28 |
| SCARB2 | Scavenger Receptor Class B Member 2 | 16.27 |
| MTOR | Mechanistic Target of Rapamycin Kinase | 16.27 |
| SOD2 | Superoxide Dismutase 2 | 16.24 |
| MMP1 | Matrix Metallopeptidase 1 | 16.21 |
| SERPINA1 | Serpin Family A Member 1 | 16.17 |
| IL2 | Interleukin 2 | 16.16 |
| MIR26B | MicroRNA 26b | 16.14 |
| CFTR | CF Transmembrane Conductance Regulator | 16.12 |
| CHMP2B | Charged Multivesicular Body Protein 2B | 16.1 |
| BCYRN1 | Brain Cytoplasmic RNA 1 | 16.1 |
| GCH1 | GTP Cyclohydrolase 1 | 16.01 |
| CALML5 | Calmodulin Like 5 | 15.89 |
| H19 | H19 Imprinted Maternally Expressed Transcript | 15.88 |
| F5 | Coagulation Factor V | 15.84 |
| PSAP | Prosaposin | 15.81 |
| NR4A2 | Nuclear Receptor Subfamily 4 Group A Member 2 | 15.8 |
| POMC | Proopiomelanocortin | 15.67 |
| ADIPOQ | Adiponectin, C1Q And Collagen Domain Containing | 15.64 |
| CD36 | CD36 Molecule | 15.57 |
| MTRR | 5-Methyltetrahydrofolate-Homocysteine Methyltransferase Reductase | 15.56 |
| MIR155 | MicroRNA 155 | 15.52 |
| CP | Ceruloplasmin | 15.47 |
| B2M | Beta-2-Microglobulin | 15.47 |
| DYNC1H1 | Dynein Cytoplasmic 1 Heavy Chain 1 | 15.41 |
| IL18 | Interleukin 18 | 15.41 |
| PRL | Prolactin | 15.39 |
| IGF1R | Insulin Like Growth Factor 1 Receptor | 15.39 |
| LTA | Lymphotoxin Alpha | 15.38 |
| DISC1 | DISC1 Scaffold Protein | 15.32 |
| CHCHD10 | Coiled-Coil-Helix-Coiled-Coil-Helix Domain Containing 10 | 15.28 |
| FASLG | Fas Ligand | 15.27 |
| BCL2 | BCL2 Apoptosis Regulator | 15.17 |
| LRP5 | LDL Receptor Related Protein 5 | 15.1 |
| FGFR3 | Fibroblast Growth Factor Receptor 3 | 15.1 |
| KIF1B | Kinesin Family Member 1B | 15.07 |
| CACNA1G | Calcium Voltage-Gated Channel Subunit Alpha1 G | 15.04 |
| AGTR1 | Angiotensin II Receptor Type 1 | 14.95 |
| SERPINI1 | Serpin Family I Member 1 | 14.94 |
| AR | Androgen Receptor | 14.86 |
| CFH | Complement Factor H | 14.8 |
| SOX2-OT | SOX2 Overlapping Transcript | 14.77 |
| CAV3 | Caveolin 3 | 14.74 |
| DDC | Dopa Decarboxylase | 14.66 |
| NR3C1 | Nuclear Receptor Subfamily 3 Group C Member 1 | 14.65 |
| FGFR2 | Fibroblast Growth Factor Receptor 2 | 14.62 |
| NRXN1 | Neurexin 1 | 14.61 |
| CR1 | Complement C3b/C4b Receptor 1 (Knops Blood Group) | 14.61 |
| MAG | Myelin Associated Glycoprotein | 14.6 |
| NLRP3 | NLR Family Pyrin Domain Containing 3 | 14.53 |
| PIK3CA | Phosphatidylinositol-4,5-Bisphosphate 3-Kinase Catalytic Subunit Alpha | 14.49 |
| C4B | Complement C4B (Chido Blood Group) | 14.47 |
| POLG | DNA Polymerase Gamma, Catalytic Subunit | 14.44 |
| LINC01080 | Long Intergenic Non-Protein Coding RNA 1080 | 14.43 |
| TYROBP | TYRO Protein Tyrosine Kinase Binding Protein | 14.42 |
| CDH1 | Cadherin 1 | 14.4 |
| EPO | Erythropoietin | 14.39 |
| ELN | Elastin | 14.38 |
| PVALB | Parvalbumin | 14.37 |
| ELANE | Elastase, Neutrophil Expressed | 14.36 |
| NDP | Norrin Cystine Knot Growth Factor NDP | 14.32 |
| INSR | Insulin Receptor | 14.28 |
| VIP | Vasoactive Intestinal Peptide | 14.27 |
| LINC01616 | Long Intergenic Non-Protein Coding RNA 1616 | 14.24 |
| PAX6 | Paired Box 6 | 14.22 |
| CYP19A1 | Cytochrome P450 Family 19 Subfamily A Member 1 | 14.21 |
| GSN | Gelsolin | 14.21 |
| TNFRSF1B | TNF Receptor Superfamily Member 1B | 14.19 |
| LINC01772 | Long Intergenic Non-Protein Coding RNA 1772 | 14.19 |
| HRAS | HRas Proto-Oncogene, GTPase | 14.18 |
| SNAP25 | Synaptosome Associated Protein 25 | 14.15 |
| SERPINC1 | Serpin Family C Member 1 | 14.13 |
| EGR2 | Early Growth Response 2 | 14.11 |
| BIN1 | Bridging Integrator 1 | 14.04 |
| RUNX1 | RUNX Family Transcription Factor 1 | 14.04 |
| SLC1A1 | Solute Carrier Family 1 Member 1 | 14.04 |
| CSTB | Cystatin B | 14 |
| REN | Renin | 13.96 |
| MBP | Myelin Basic Protein | 13.95 |
| TNFSF11 | TNF Superfamily Member 11 | 13.91 |
| GARS | Glycyl-TRNA Synthetase | 13.9 |
| HSPD1 | Heat Shock Protein Family D (Hsp60) Member 1 | 13.89 |
| IL12B | Interleukin 12B | 13.83 |
| LCAT | Lecithin-Cholesterol Acyltransferase | 13.81 |
| LIPA | Lipase A, Lysosomal Acid Type | 13.81 |
| UBQLN2 | Ubiquilin 2 | 13.78 |
| AGT | Angiotensinogen | 13.74 |
| FGFR1 | Fibroblast Growth Factor Receptor 1 | 13.71 |
| IL23R | Interleukin 23 Receptor | 13.69 |
| HAR1A | Highly Accelerated Region 1A | 13.62 |
| HSPG2 | Heparan Sulfate Proteoglycan 2 | 13.61 |
| CHRNA4 | Cholinergic Receptor Nicotinic Alpha 4 Subunit | 13.59 |
| BRCA1 | BRCA1 DNA Repair Associated | 13.58 |
| HAR1B | Highly Accelerated Region 1B | 13.51 |
| LRP1-AS | LRP1 Antisense RNA | 13.48 |
| THBD | Thrombomodulin | 13.47 |
| TGFB2 | Transforming Growth Factor Beta 2 | 13.45 |
| MIR363 | MicroRNA 363 | 13.45 |
| IL13 | Interleukin 13 | 13.43 |
| G6PD | Glucose-6-Phosphate Dehydrogenase | 13.36 |
| HMGCR | 3-Hydroxy-3-Methylglutaryl-CoA Reductase | 13.33 |
| MIR511 | MicroRNA 511 | 13.32 |
| NFKB1 | Nuclear Factor Kappa B Subunit 1 | 13.32 |
| FGF23 | Fibroblast Growth Factor 23 | 13.25 |
| JUP | Junction Plakoglobin | 13.24 |
| GNB3 | G Protein Subunit Beta 3 | 13.21 |
| SNHG3 | Small Nucleolar RNA Host Gene 3 | 13.21 |
| CYBA | Cytochrome B-245 Alpha Chain | 13.2 |
| CRYAB | Crystallin Alpha B | 13.17 |
| ATM | ATM Serine/Threonine Kinase | 13.17 |
| PDGFRB | Platelet Derived Growth Factor Receptor Beta | 13.16 |
| APOC3 | Apolipoprotein C3 | 13.16 |
| PAX2 | Paired Box 2 | 13.13 |
| TNFRSF11A | TNF Receptor Superfamily Member 11a | 13.13 |
| HEXB | Hexosaminidase Subunit Beta | 13.12 |
| CCL3 | C-C Motif Chemokine Ligand 3 | 13.11 |
| SELP | Selectin P | 13.11 |
| BRAF | B-Raf Proto-Oncogene, Serine/Threonine Kinase | 13.11 |
| PIK3R1 | Phosphoinositide-3-Kinase Regulatory Subunit 1 | 13.04 |
| MBL2 | Mannose Binding Lectin 2 | 13.04 |
| TERT | Telomerase Reverse Transcriptase | 13.04 |
| MEFV | MEFV Innate Immuity Regulator, Pyrin | 13.03 |
| GSTM1 | Glutathione S-Transferase Mu 1 | 13 |
| GRM1 | Glutamate Metabotropic Receptor 1 | 12.97 |
| HSPB1 | Heat Shock Protein Family B (Small) Member 1 | 12.97 |
| IL17A | Interleukin 17A | 12.96 |
| CASR | Calcium Sensing Receptor | 12.95 |
| ATP7A | ATPase Copper Transporting Alpha | 12.9 |
| NCF2 | Neutrophil Cytosolic Factor 2 | 12.88 |
| COL6A1 | Collagen Type VI Alpha 1 Chain | 12.86 |
| COG2 | Component Of Oligomeric Golgi Complex 2 | 12.85 |
| MYC | MYC Proto-Oncogene, BHLH Transcription Factor | 12.77 |
| KIF5A | Kinesin Family Member 5A | 12.74 |
| WT1 | WT1 Transcription Factor | 12.73 |
| NPPA | Natriuretic Peptide A | 12.72 |
| NPPB | Natriuretic Peptide B | 12.72 |
| FN1 | Fibronectin 1 | 12.71 |
| CNTNAP2 | Contactin Associated Protein Like 2 | 12.71 |
| PHYH | Phytanoyl-CoA 2-Hydroxylase | 12.69 |
| CCND1 | Cyclin D1 | 12.67 |
| IGF2 | Insulin Like Growth Factor 2 | 12.66 |
| C3 | Complement C3 | 12.64 |
| CNR1 | Cannabinoid Receptor 1 | 12.61 |
| MTR | 5-Methyltetrahydrofolate-Homocysteine Methyltransferase | 12.6 |
| PITX3 | Paired Like Homeodomain 3 | 12.57 |
| ESR2 | Estrogen Receptor 2 | 12.53 |
| SRC | SRC Proto-Oncogene, Non-Receptor Tyrosine Kinase | 12.53 |
| KCTD7 | Potassium Channel Tetramerization Domain Containing 7 | 12.52 |
| EDN3 | Endothelin 3 | 12.51 |
| SLC6A2 | Solute Carrier Family 6 Member 2 | 12.49 |
| NLRP1 | NLR Family Pyrin Domain Containing 1 | 12.48 |
| DAOA | D-Amino Acid Oxidase Activator | 12.47 |
| GNAS | GNAS Complex Locus | 12.47 |
| ERCC2 | ERCC Excision Repair 2, TFIIH Core Complex Helicase Subunit | 12.43 |
| TBX1 | T-Box 1 | 12.4 |
| MIF | Macrophage Migration Inhibitory Factor | 12.36 |
| TGFBR2 | Transforming Growth Factor Beta Receptor 2 | 12.36 |
| LIPC | Lipase C, Hepatic Type | 12.36 |
| TTN | Titin | 12.35 |
| ITIH4 | Inter-Alpha-Trypsin Inhibitor Heavy Chain 4 | 12.34 |
| DRD5 | Dopamine Receptor D5 | 12.34 |
| DRD1 | Dopamine Receptor D1 | 12.34 |
| SYNJ1 | Synaptojanin 1 | 12.33 |
| TFRC | Transferrin Receptor | 12.32 |
| NF1 | Neurofibromin 1 | 12.24 |
| NAGLU | N-Acetyl-Alpha-Glucosaminidase | 12.24 |
| CCL5 | C-C Motif Chemokine Ligand 5 | 12.23 |
| CXCR4 | C-X-C Motif Chemokine Receptor 4 | 12.22 |
| RTN4R | Reticulon 4 Receptor | 12.22 |
| DTNBP1 | Dystrobrevin Binding Protein 1 | 12.2 |
| CCK | Cholecystokinin | 12.18 |
| COL1A1 | Collagen Type I Alpha 1 Chain | 12.17 |
| APC | APC Regulator of WNT Signaling Pathway | 12.16 |
| FBN1 | Fibrillin 1 | 12.16 |
| IRS1 | Insulin Receptor Substrate 1 | 12.16 |
| RBPJ | Recombination Signal Binding Protein for Immunoglobulin Kappa J Region | 12.16 |
| GGT1 | Gamma-Glutamyltransferase 1 | 12.15 |
| HP | Haptoglobin | 12.15 |
| NAGA | Alpha-N-Acetylgalactosaminidase | 12.12 |
| NDUFV2 | NADH:Ubiquinone Oxidoreductase Core Subunit V2 | 12.11 |
| VIM | Vimentin | 12.1 |
| ECE1 | Endothelin Converting Enzyme 1 | 12.1 |
| CAV1 | Caveolin 1 | 12.08 |
| TPO | Thyroid Peroxidase | 12.08 |
| NRTN | Neurturin | 12.06 |
| BAX | BCL2 Associated X, Apoptosis Regulator | 12.05 |
| STAT1 | Signal Transducer and Activator Of Transcription 1 | 12.03 |
| SPP1 | Secreted Phosphoprotein 1 | 12.02 |
| ADA | Adenosine Deaminase | 11.99 |
| GSTP1 | Glutathione S-Transferase Pi 1 | 11.99 |
| TSC2 | TSC Complex Subunit 2 | 11.97 |
| HTR1A | 5-Hydroxytryptamine Receptor 1A | 11.97 |
| CCL11 | C-C Motif Chemokine Ligand 11 | 11.96 |
| SMARCA2 | SWI/SNF Related, Matrix Associated, Actin Dependent Regulator of Chromatin, Subfamily A, Member 2 | 11.95 |
| CCR6 | C-C Motif Chemokine Receptor 6 | 11.93 |
| TPH2 | Tryptophan Hydroxylase 2 | 11.93 |
| HTR6 | 5-Hydroxytryptamine Receptor 6 | 11.89 |
| SCO1 | SCO Cytochrome C Oxidase Assembly Protein 1 | 11.89 |
| YWHAQ | Tyrosine 3-Monooxygenase/Tryptophan 5-Monooxygenase Activation Protein Theta | 11.85 |
| ABL1 | ABL Proto-Oncogene 1, Non-Receptor Tyrosine Kinase | 11.85 |
| MLYCD | Malonyl-CoA Decarboxylase | 11.84 |
| DNAJC13 | DnaJ Heat Shock Protein Family (Hsp40) Member C13 | 11.83 |
| STUB1 | STIP1 Homology And U-Box Containing Protein 1 | 11.82 |
| MMP13 | Matrix Metallopeptidase 13 | 11.82 |
| SELE | Selectin E | 11.8 |
| HLA-DPB1 | Major Histocompatibility Complex, Class II, DP Beta 1 | 11.79 |
| PDGFRA | Platelet Derived Growth Factor Receptor Alpha | 11.77 |
| SFTPC | Surfactant Protein C | 11.76 |
| STK11 | Serine/Threonine Kinase 11 | 11.75 |
| LMX1B | LIM Homeobox Transcription Factor 1 Beta | 11.74 |
| APOL1 | Apolipoprotein L1 | 11.74 |
| CACNA1A | Calcium Voltage-Gated Channel Subunit Alpha1 A | 11.69 |
| SMAD3 | SMAD Family Member 3 | 11.68 |
| CHRNB2 | Cholinergic Receptor Nicotinic Beta 2 Subunit | 11.67 |
| MEGF10 | Multiple EGF Like Domains 10 | 11.66 |
| SGCE | Sarcoglycan Epsilon | 11.66 |
| F13A1 | Coagulation Factor XIII A Chain | 11.66 |
| NFE2L2 | Nuclear Factor, Erythroid 2 Like 2 | 11.65 |
| LOC110806262 | Solute Carrier Family 6 Member 4 Gene Promoter | 11.63 |
| JUN | Jun Proto-Oncogene, AP-1 Transcription Factor Subunit | 11.63 |
| GH1 | Growth Hormone 1 | 11.61 |
| CDKN1A | Cyclin Dependent Kinase Inhibitor 1A | 11.6 |
| PRKCSH | Protein Kinase C Substrate 80K-H | 11.6 |
| NDRG1 | N-Myc Downstream Regulated 1 | 11.59 |
| PRODH | Proline Dehydrogenase 1 | 11.53 |
| LPA | Lipoprotein(A) | 11.52 |
| SAA1 | Serum Amyloid A1 | 11.51 |
| ENO2 | Enolase 2 | 11.48 |
| SMAD4 | SMAD Family Member 4 | 11.41 |
| DLD | Dihydrolipoamide Dehydrogenase | 11.41 |
| GAD1 | Glutamate Decarboxylase 1 | 11.4 |
| MIR29C | MicroRNA 29c | 11.39 |
| CBS | Cystathionine-Beta-Synthase | 11.38 |
| SMN1 | Survival Of Motor Neuron 1, Telomeric | 11.37 |
| TRPM7 | Transient Receptor Potential Cation Channel Subfamily M Member 7 | 11.37 |
| PLAT | Plasminogen Activator, Tissue Type | 11.35 |
| RBP4 | Retinol Binding Protein 4 | 11.34 |
| LTF | Lactotransferrin | 11.34 |
| HLA-DQA1 | Major Histocompatibility Complex, Class II, DQ Alpha 1 | 11.33 |
| NR3C2 | Nuclear Receptor Subfamily 3 Group C Member 2 | 11.3 |
| PON2 | Paraoxonase 2 | 11.3 |
| PSNP3 | Supranuclear Palsy, Progressive, 3 | 11.27 |
| ADAR | Adenosine Deaminase RNA Specific | 11.22 |
| TPH1 | Tryptophan Hydroxylase 1 | 11.22 |
| PSNP2 | Supranuclear Palsy, Progressive, 2 | 11.22 |
| CXCL12 | C-X-C Motif Chemokine Ligand 12 | 11.17 |
| SLC11A1 | Solute Carrier Family 11 Member 1 | 11.15 |
| XRCC1 | X-Ray Repair Cross Complementing 1 | 11.15 |
| IL21 | Interleukin 21 | 11.14 |
| MED12 | Mediator Complex Subunit 12 | 11.14 |
| RAB7A | RAB7A, Member RAS Oncogene Family | 11.13 |
| DAO | D-Amino Acid Oxidase | 11.12 |
| PAFAH1B1 | Platelet Activating Factor Acetylhydrolase 1b Regulatory Subunit 1 | 11.12 |
| LRP2 | LDL Receptor Related Protein 2 | 11.12 |
| VCAM1 | Vascular Cell Adhesion Molecule 1 | 11.11 |
| CRHR1 | Corticotropin Releasing Hormone Receptor 1 | 11.09 |
| PMPCA | Peptidase, Mitochondrial Processing Alpha Subunit | 11.09 |
| GHRL | Ghrelin and Obestatin Prepropeptide | 11.07 |
| CSF3 | Colony Stimulating Factor 3 | 11.07 |
| CHKB | Choline Kinase Beta | 11.07 |
| ENPP1 | Ectonucleotide Pyrophosphatase/Phosphodiesterase 1 | 11.06 |
| CLN3 | CLN3 Lysosomal/Endosomal Transmembrane Protein, Battenin | 11.06 |
| HTRA2 | HtrA Serine Peptidase 2 | 11.03 |
| ITGB3 | Integrin Subunit Beta 3 | 11.02 |
| CCR1 | C-C Motif Chemokine Receptor 1 | 10.99 |
| IL6R | Interleukin 6 Receptor | 10.96 |
| COL17A1 | Collagen Type XVII Alpha 1 Chain | 10.94 |
| DMD | Dystrophin | 10.94 |
| GSTT1 | Glutathione S-Transferase Theta 1 | 10.93 |
| CYP17A1 | Cytochrome P450 Family 17 Subfamily A Member 1 | 10.93 |
| ADRB2 | Adrenoceptor Beta 2 | 10.93 |
| TIMP1 | TIMP Metallopeptidase Inhibitor 1 | 10.92 |
| MT-CYB | Mitochondrially Encoded Cytochrome B | 10.91 |
| NAT2 | N-Acetyltransferase 2 | 10.91 |
| FGF2 | Fibroblast Growth Factor 2 | 10.9 |
| FGF20 | Fibroblast Growth Factor 20 | 10.9 |
| MAP2K1 | Mitogen-Activated Protein Kinase Kinase 1 | 10.89 |
| XDH | Xanthine Dehydrogenase | 10.83 |
| ASAH1 | N-Acylsphingosine Amidohydrolase 1 | 10.82 |
| VPS13C | Vacuolar Protein Sorting 13 Homolog C | 10.81 |
| CYP27A1 | Cytochrome P450 Family 27 Subfamily A Member 1 | 10.81 |
| CREBBP | CREB Binding Protein | 10.78 |
| BMP6 | Bone Morphogenetic Protein 6 | 10.76 |
| ACTA2 | Actin Alpha 2, Smooth Muscle | 10.76 |
| HIF1A | Hypoxia Inducible Factor 1 Subunit Alpha | 10.73 |
| GRIA3 | Glutamate Ionotropic Receptor AMPA Type Subunit 3 | 10.71 |
| GOSR2 | Golgi SNAP Receptor Complex Member 2 | 10.69 |
| NTRK3 | Neurotrophic Receptor Tyrosine Kinase 3 | 10.66 |
| BGLAP | Bone Gamma-Carboxyglutamate Protein | 10.66 |
| PTH | Parathyroid Hormone | 10.65 |
| LRSAM1 | Leucine Rich Repeat and Sterile Alpha Motif Containing 1 | 10.63 |
| SDHB | Succinate Dehydrogenase Complex Iron Sulfur Subunit B | 10.62 |
| GPT | Glutamic--Pyruvic Transaminase | 10.61 |
| PLA2G7 | Phospholipase A2 Group VII | 10.61 |
| CDKN1B | Cyclin Dependent Kinase Inhibitor 1B | 10.58 |
| RETN | Resistin | 10.57 |
| HRH2 | Histamine Receptor H2 | 10.54 |
| F11 | Coagulation Factor XI | 10.54 |
| IFNGR1 | Interferon Gamma Receptor 1 | 10.52 |
| MT-ND4 | Mitochondrially Encoded NADH:Ubiquinone Oxidoreductase Core Subunit 4 | 10.51 |
| MIR195 | MicroRNA 195 | 10.51 |
| SAG | S-Antigen Visual Arrestin | 10.51 |
| COL4A4 | Collagen Type IV Alpha 4 Chain | 10.49 |
| VDAC1 | Voltage Dependent Anion Channel 1 | 10.46 |
| KITLG | KIT Ligand | 10.42 |
| ADORA2A | Adenosine A2a Receptor | 10.39 |
| ATP2C1 | ATPase Secretory Pathway Ca2+ Transporting 1 | 10.36 |
| EPOR | Erythropoietin Receptor | 10.36 |
| GNPTAB | N-Acetylglucosamine-1-Phosphate Transferase Subunits Alpha and Beta | 10.35 |
| SHANK3 | SH3 And Multiple Ankyrin Repeat Domains 3 | 10.34 |
| OGG1 | 8-Oxoguanine DNA Glycosylase | 10.3 |
| F7 | Coagulation Factor VII | 10.28 |
| SDHD | Succinate Dehydrogenase Complex Subunit D | 10.28 |
| DES | Desmin | 10.26 |
| PRICKLE1 | Prickle Planar Cell Polarity Protein 1 | 10.26 |
| F12 | Coagulation Factor XII | 10.24 |
| PTCH1 | Patched 1 | 10.22 |
| CDKN3 | Cyclin Dependent Kinase Inhibitor 3 | 10.21 |
| GP1BA | Glycoprotein Ib Platelet Subunit Alpha | 10.21 |
| PPARA | Peroxisome Proliferator Activated Receptor Alpha | 10.21 |
| SPTLC1 | Serine Palmitoyltransferase Long Chain Base Subunit 1 | 10.21 |
| MSH2 | MutS Homolog 2 | 10.2 |
| DNM2 | Dynamin 2 | 10.2 |
| MT-ATP6 | Mitochondrially Encoded ATP Synthase Membrane Subunit 6 | 10.2 |
| TNNI3 | Troponin I3, Cardiac Type | 10.17 |
| LEPR | Leptin Receptor | 10.16 |
| ZAP70 | Zeta Chain of T Cell Receptor Associated Protein Kinase 70 | 10.15 |
| PDCD1 | Programmed Cell Death 1 | 10.14 |
| MAPK14 | Mitogen-Activated Protein Kinase 14 | 10.13 |
| PRKCD | Protein Kinase C Delta | 10.1 |
| F3 | Coagulation Factor III, Tissue Factor | 10.09 |
| SEMA3C | Semaphorin 3C | 10.08 |
| CALB2 | Calbindin 2 | 10.08 |
| MDM2 | MDM2 Proto-Oncogene | 10.08 |
| MOBP | Myelin Associated Oligodendrocyte Basic Protein | 10.07 |
| GALC | Galactosylceramidase | 10.07 |
| PCNT | Pericentrin | 10.06 |
| ENG | Endoglin | 10.05 |
| NEFH | Neurofilament Heavy | 10.05 |
| GJA1 | Gap Junction Protein Alpha 1 | 10.01 |

TABLE S10: 34 shared targets for at least 5 herbs and AD.

| Targets | Numbers of herbs | Herbs | | | | | | |
| --- | --- | --- | --- | --- | --- | --- | --- | --- |
| EGFR | 5 | CPA | CA | LJ | HC | GJ |  |  |
| ESR1 |  | AP | CPA | CC | LJ | GJ |  |  |
| ESR2 |  | AP | CPA | CC | LJ | GJ |  |  |
| VEGFA |  | CPA | CA | LJ | HC | GJ |  |  |
| ACHE | 6 | AP | CPA | CA | LJ | HC | GJ |  |
| BCL2 |  | AP | CPA | CA | LJ | HC | GJ |  |
| CASP8 |  | CPA | CC | CA | LJ | HC | GJ |  |
| CAV1 |  | CPA | CC | CA | LJ | HC | GJ |  |
| CCND1 |  | AP | CPA | CA | LJ | HC | GJ |  |
| CRP |  | CPA | CC | CA | LJ | HC | GJ |  |
| CTSD |  | CPA | CC | CA | LJ | HC | GJ |  |
| ERBB2 |  | CPA | CC | CA | LJ | HC | GJ |  |
| GSTM1 |  | CPA | CC | CA | LJ | HC | GJ |  |
| GSTP1 |  | CPA | CC | CA | LJ | HC | GJ |  |
| HIF1A |  | CPA | CC | CA | LJ | HC | GJ |  |
| HSPB1 |  | CPA | CC | CA | LJ | HC | GJ |  |
| ICAM1 |  | CPA | CC | CA | LJ | HC | GJ |  |
| IGF2 |  | CPA | CC | CA | LJ | HC | GJ |  |
| MYC |  | CPA | CC | CA | LJ | HC | GJ |  |
| NFE2L2 |  | CPA | CC | CA | LJ | HC | GJ |  |
| NOS3 |  | CPA | CC | CA | LJ | HC | GJ |  |
| NQO1 |  | CPA | CC | CA | LJ | HC | GJ |  |
| PLAU |  | CPA | CC | CA | LJ | HC | GJ |  |
| PON1 |  | CPA | CC | CA | LJ | HC | GJ |  |
| SELE |  | CPA | CC | CA | LJ | HC | GJ |  |
| VCAM1 |  | CPA | CC | CA | LJ | HC | GJ |  |
| AR | 7 | AP | CPA | CC | CA | LJ | HC | GJ |
| CASP3 |  | AP | CPA | CC | CA | LJ | HC | GJ |
| CASP9 |  | AP | CPA | CC | CA | LJ | HC | GJ |
| F7 |  | AP | CPA | CC | CA | LJ | HC | GJ |
| IL6 |  | AP | CPA | CC | CA | LJ | HC | GJ |
| NR3C2 |  | AP | CPA | CC | CA | LJ | HC | GJ |
| PPARG |  | AP | CPA | CC | CA | LJ | HC | GJ |
| PTGS1 |  | AP | CPA | CC | CA | LJ | HC | GJ |
